# *Urtica dioica* L. Phytochemistry, Green Extraction Techniques, Molecular Mechanisms, and Gene Expression Modulation: A Comprehensive Review

**DOI:** 10.3390/antiox15080928

**Published:** 2026-07-27

**Authors:** Noor Alriyahi, Ammar Badran Ramddan, Nawfal Alhelfi, Asad Abbas, Ralf Weiskirchen, Farhang Hameed Awlqadr, Ghalia Arshad, Hassan Raza

**Affiliations:** 1 Department of Food Science, College of Agriculture, University of Basrah, Basrah 61004, Iraq; noor.abdulamer@uobasrah.edu.iq (N.A.); nawfal.hussain@uobasrah.edu.iq (N.A.); 2 Department of Human Nutrition, Faculty of Food Science and Nutrition, Bahauddin Zakariya University, Multan 60800, Pakistan; asadabbaskhichi@gmail.com (A.A.); ghaliaarshad0@gmail.com (G.A.); 3 Institute of Molecular Pathobiochemistry, Experimental Gene Therapy and Clinical Chemistry (IFMPEGKC), RWTH University Hospital Aachen, D-52074 Aachen, Germany; 4 Department of Food Science and Technology, Faculty of Agriculture, University of Tabriz, Tabriz 51369, Iran; farhang.hamid.a@spu.edu.iq; 5 Food Science and Quality Control, Halabja Technical College, Sulaimani Polytechnic University, Sulaymaniyah 46001, Iraq; 6 Department of Food Science & Technology, Faculty of Food Science and Nutrition, Bahauddin Zakariya University, Multan 60800, Pakistan; sh.raza40@yahoo.com

**Keywords:** *Urtica dioica*, stinging nettle, phytochemistry, green extraction, antioxidant, anti-inflammatory, gene modulation, NADES, nutraceuticals

## Abstract

*Urtica dioica* L. (stinging nettle) is a perennial herb with a long ethnomedicinal history and diverse pharmacological potential. This comprehensive review consolidates current knowledge on its phytochemistry, extraction technologies, bioactivities, and molecular mechanisms. However, recent reviews have generally addressed these aspects separately, and an integrated assessment linking green extraction technologies and phytochemical profiles to molecular mechanisms and gene expression modulation is still lacking. *U. dioica* contains abundant polyphenols (rutin, quercetin, kaempferol, and chlorogenic acid), sterols (β-sitosterol and stigmasterol), vitamins, carotenoids, and the antiviral lectin *Urtica dioica* agglutinin (UDA). Advances in green extraction technologies, such as ultrasound-assisted extraction, microwave-assisted extraction (MAE), pressurized liquid extraction, and natural deep eutectic solvent (NADES)-based systems, have significantly improved yield, purity, and environmental sustainability compared to conventional maceration and Soxhlet methods. Comprehensive chromatographic and spectroscopic profiling (HPLC, GC–MS, FTIR, NMR, and LC–MS/MS) has established detailed chemical fingerprints linking bioactive constituents to antioxidant, anti-inflammatory, antimicrobial, and antiviral properties. Mechanistic studies reveal that *U. dioica* exerts its therapeutic effects through modulation of oxidative stress, inhibition of the NF-κB and COX-2 pathways, enhancement of endogenous antioxidant enzymes, and regulation of apoptotic gene expression. Moreover, NADES–MAE extracts demonstrate potential as sustainable, high-efficacy formulations for nutraceutical and cosmetic applications. Despite extensive preclinical evidence, clinical standardization and dosage optimization remain major challenges. This review underscores *U. dioica* as a multifunctional medicinal plant with significant promise for next-generation phytotherapeutics and molecular nutrition.

## 1. Introduction

*Urtica dioica* L., commonly known as stinging nettle, is a herbaceous perennial plant of the Urticaceae family. The plant, native to Eurasia, but now common in Europe, Asia, North Africa, and North America, has long been used for culinary and medical purposes. *U. dioica*, which is a stinging plant that grows well in damp, nutrient-rich settings, has historically been used in traditional medicine to treat various diseases including joint pain, skin irritation, allergies, and urinary tract problems [1]. Although often viewed as a typical weed in most of the world, stinging nettle has a rich ethnobotanical history and pharmacological potential that has drawn increased scientific attention over the last few years.

The growing need for safe, natural, and effective treatments has prompted the reconsideration of medicinal plants such as *U. dioica* in modern pharmacology and nutritional sciences. The plant has a large concentration of bioactive constituents such as flavonoids, phenolic acids, sterols, vitamins, minerals, lignans, and fatty acids in various parts of the plant such as leaves, stems, roots, and seeds [2]. These compounds have been linked to various therapeutic activities, including antioxidant, anti-inflammatory, antimicrobial, antihyperlipidemic, and antidiabetic effects. Additionally, *U. dioica* roots are used traditionally to treat lower urinary tract symptoms associated with benign prostatic hyperplasia (BPH), and recent research shows that they may inhibit 5-alpha reductase and reduce inflammation in prostate tissue [2,3].

Phytochemical research on *U. dioica* has also highlighted the presence of particular compounds like kaempferol, rutin, chlorogenic acid, β-sitosterol, and *U. dioica* agglutinin (UDA), a lectin reported to have immunomodulatory and antiviral effects [4]. The findings support the development of nutraceuticals, functional foods, and herbal preparations based on nettle. However, the success of these products is closely related to the quality of their bioactive extracts, which may differ considerably depending on the extraction technique. Both traditional methods, which include maceration and Soxhlet extraction, and modern green techniques, including microwave-assisted extraction (MAE), ultrasound-assisted extraction (UAE), and natural deep eutectic solvent (NADES) systems, have been compared [5]. Recent investigations have shown that NADES in combination with MAE can produce extracts with enhanced antioxidant and antimicrobial properties and provide sustainable alternatives for large-scale phytochemical extraction [6].

In addition, *U. dioica* has shown beneficial effects at the molecular level. In vitro studies indicate that it may enhance the apoptotic potential of chemotherapy agents like cisplatin, suggesting potential applicability in oncology. For example, a leaf infusion of *U. dioica* in conjunction with cisplatin has been reported to inhibit the growth of ovarian cancer cells, increase apoptosis-related caspase protein activity, and reduce the migration and invasion of cancer cells [7]. These findings highlight the potential of nettle to regulate gene expression, signaling pathways, and epigenetic mechanisms, which contributes to the emerging interest in nettle as a molecular therapeutic agent.

Although *U. dioica* has extensive traditional use and promising preliminary results, its application in clinical practice is limited by numerous obstacles such as variability in extract composition, non-standardized dosages, and a limited number of human studies. The therapeutic success of nettle-based therapies depends heavily on the plant parts used, the harvest season, geographical origin, and processing methods. Furthermore, although numerous in vitro and animal studies indicate significant bioactivity, they have not been sufficiently validated in human populations [8]. It is therefore important to address these research gaps by undertaking rigorous clinical trials, molecular research, and further development of standardized extraction methods, which are important for unlocking the complete pharmacological potential of this versatile plant. The purpose of this review is to critically examine *U. dioica* by analyzing its botanical properties, ethnomedical applications, and its applicability in modern pharmacology. Particularly, it will assess extraction procedures for obtaining its bioactive compounds, examine its phytochemical composition and their relationship to therapeutic properties, and discuss the molecular and genetic pathways that underlie its biological actions. Furthermore, this review evaluates clinical and preclinical data, safety considerations, and future prospects for the use of *U. dioica* in both standardized medical and nutraceutical applications.

## 2. Search Methodology

This review was conducted using a structured literature search strategy to identify and collect relevant studies on *Urtica dioica* covering its phytochemistry, morphology, clinical applications, and pharmacological properties. Data were collected from PubMed, Google Scholar, Scopus, ScienceDirect, and Web of Science, covering studies published up to October 2025. Search terms included “*Urtica dioica*”, “stinging nettle”, “extraction methods”, “phytochemistry”, “NADES”, “biological activity”, “leaves”, “roots”, “seeds”, “anti-inflammatory”, and “clinical applications”. Boolean operators (AND, OR) were used to refine the search results.

## 3. *Urtica dioica* History and Botanical Description

*U. dioica*, also referred to as stinging nettle, is a perennial, herbaceous plant that belongs to the family Urticaceae. Its use dates back thousands of years, giving it a long historical reputation in both classical and contemporary pharmacopoeias. Nettle is traditionally used for food, textiles, and as medicine across different continents. The use of nettle fibers in Europe can be traced back to the Bronze Age, when they were used to create clothing. The ancient Greeks and Romans were also aware of its medicinal properties, as they used nettle extracts to treat arthritis and increase circulation, which was attributed to the irritant properties of the plant [9].

**Figure 1:**
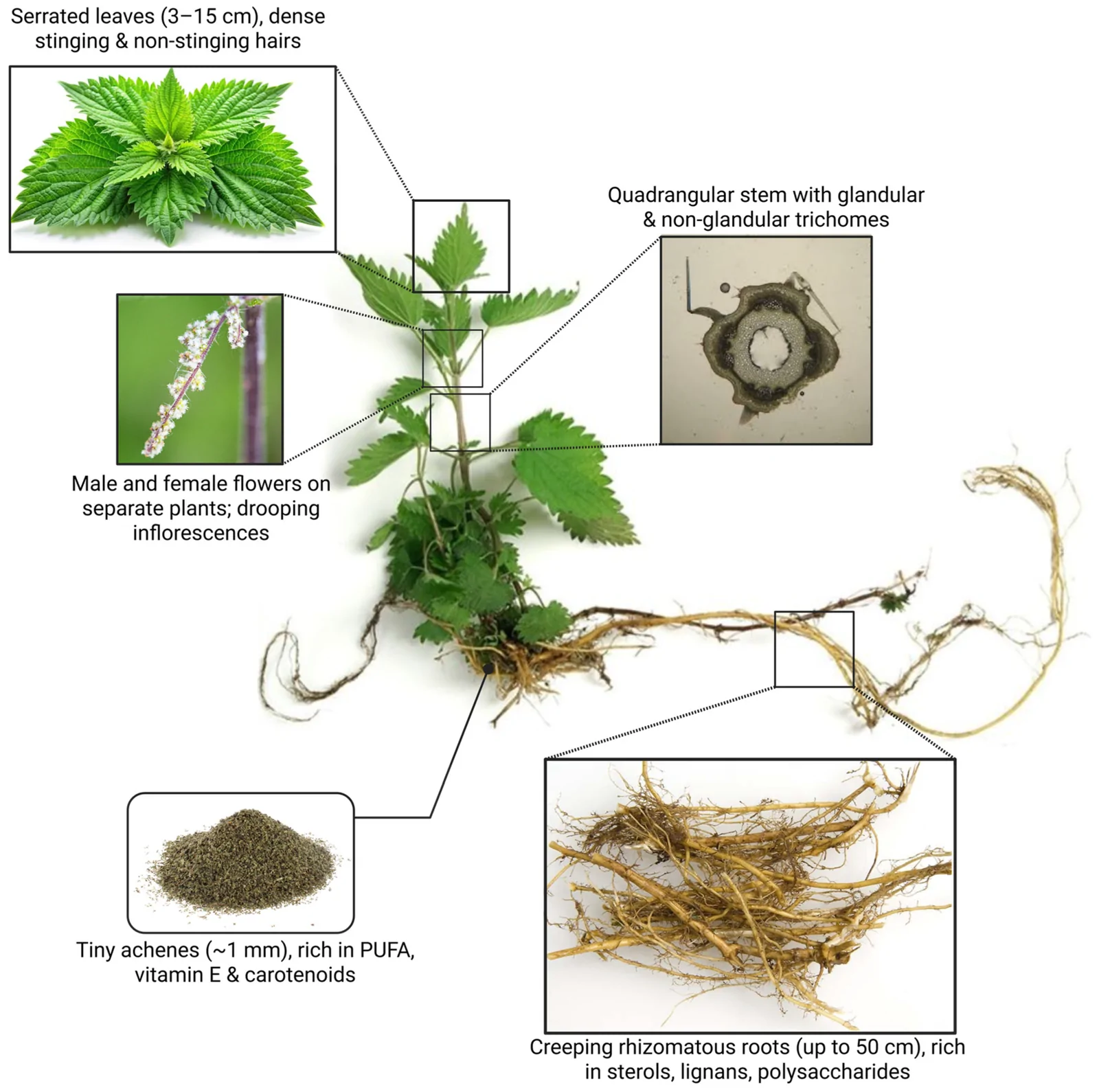
Key morphological features of *U. dioica*, including serrated leaves with stinging and non-stinging hairs, quadrangular stems with glandular trichomes, drooping unisexual inflorescences, tiny nutrient-rich achenes, and creeping rhizomatous roots rich in sterols and lignans. Created in BioRender. Awlqadr, F. (accessed on 14 July 2026), https://BioRender.com/s4kwi4t.

*U. dioica* is botanically dioecious, meaning that male and female flowers occur on different plants. It is usually found in temperate regions of Europe, Asia, North America, and North Africa, in nitrogen-rich soils near water bodies, along roadsides, and in meadows. The plant heigh typically ranges from 50 cm to more than 2 m, depending on the environment. It has quadrangular stems covered with both stinging and non-stinging hairs. The stinging trichomes contain histamine, acetylcholine, serotonin, and formic acid, which cause skin irritation upon contact [10]. Leaves are opposite, ovate to lanceolate, sharply toothed along the margins, and density covered with glandular and non-glandular hairs. The size of leaves varies, but they are usually 3–15 cm in length and 1–5 cm in width. The upper surface of the leaf is dark green, while the lower side is lighter in color. The flowers are small, greenish, and unisexual, and are borne in long, dangling clusters in the axils of upper leaves. Male flowers release their pollen explosively to optimize the fertilization process, whereas female flowers develop into small, one-seeded achenes [11]. The rhizomatous root system is extensive. It creates creeping rhizomes, which enable the plant to spread efficiently. The rhizomes may extend horizontally up to 50 cm and are normally yellowish-brown. *U. dioica* roots and rhizomes contain high levels of sterols, lignans, and polysaccharides and have been used as a remedy for benign prostatic hyperplasia [4,12]. Figure 1 highlights the key morphological features of *U. dioica* described in the text, including serrated leaves with stinging and non-stinging trichomes, drooping unisexual inflorescences, quadrangular stems with glandular hairs, creeping rhizomatous roots rich in sterols, small nutrient-rich achenes, and polysaccharides.

Modern pharmacognosy continues to explore the root extract’s therapeutic potential, especially for its anti-androgenic and anti-inflammatory effects. *U. dioica* seeds are tiny, brownish, and ovoid, measuring around 1 mm in diameter. Despite their small size, they are nutritionally dense, with rich in polyunsaturated fatty acids (PUFA), carotenoids, and vitamin E. Traditionally, seeds were utilized as tonics to support kidney function and physical endurance, and recent studies have also begun investigating their antioxidant properties [13].

Each part of *U. dioica* serves distinct nutritional and medicinal functions, with the leaves being the most commonly used, rich in vitamins A and C, flavonoids, chlorophyll, and minerals like calcium, iron, and magnesium. Leaf infusions are used for hematopoietic, diuretic, and antirheumatic purposes. Stems, although pharmacologically less active, have traditionally been used in fiber production. Roots contain potent bioactive compounds for prostate and urinary health, while seeds are used to support hormonal vitality and balance [10,14].

Modern studies confirm that the entire plant (leaves, roots, stems, and seeds) contains a broad spectrum of bioactive compounds including polyphenols, tannins, sterols, lignans, essential amino acids, and terpenoids. Moreover, growing interest in green extraction technologies, like MAE, has improved the purity and yield of these compounds, reinforcing the importance of each morphological component [15]. In conclusion, the comprehensive morphological structure of *U. dioica*, coupled with its versatile applications and rich history, underscores its prominence as a multifunctional medicinal plant. Its distinct parts (leaf, root, stem, and seed) each offer unique nutritional or therapeutic value, making it an important subject of both historical reverence and modern scientific inquiry.

## 4. Chemical Composition and Phytochemical Profiling of *Urtica dioica*

*U. dioica* has a remarkably diverse and complex chemical composition, reflecting the plant’s rich profile of secondary metabolites that contribute to its broad therapeutic potential. Detailed phytochemical studies show that nettle contains abundant polyphenols, particularly flavonoids such as rutin, kaempferol, quercetin, and isorhamnetin derivatives, along with phenolic acids including chlorogenic, ferulic, caffeic, and 5-caffeoylquinic acids. Together, these compounds form the biochemical foundation of its strong anti-inflammatory and antioxidant activities [16]. Chromatographic analyses (UPLC-MS, HPLC-DAD) confirm that the leaves contain the highest concentrations of phenolics, with several studies identifying rutin, chlorogenic acid, and quercetin as major constituents in hydroalcoholic extracts [17].

In addition to polyphenols, *U. dioica* also contains significant amounts of volatile and terpenoid compounds such as α-terpineol, β-caryophyllene, phytol, and 1-octen-3-ol, which have been identified by GC–MS in supercritical CO_2_ extracts or hydrodistilled fractions and contribute to the plant’s dermoprotective, antimicrobial, and anti-inflammatory effects [2]. Meanwhile, the seeds and roots are particularly rich in triterpenoids and phytosterols, including stigmasterol, β-sitosterol, β-amyrin, campesterol, and lupeol. These compounds play important roles in prostate health, lipid regulation, and anti-inflammatory activity [18]. Pressurized liquid extraction has also demonstrated a high recovery of sterols from the roots, yielding β-sitosterol levels of up to 50 mg/100 g, which exceeds those obtained using conventional extraction techniques.

Seeds additionally contain nutritionally relevant polyunsaturated fatty acids, notably α-linolenic and linoleic acids, which contribute to anti-inflammatory and cardiometabolic benefits. Beyond lipids and phenolics, *U. dioica* is also a notable source of vitamins (A, C, and B-complex), minerals (iron, calcium, and magnesium), chlorophylls, carotenoids, and essential amino acids. Studies examining the fatty acid profile of *U. dioica* across different plant parts show variations in saturated, monounsaturated, and polyunsaturated fatty acids, with mature leaves being rich in α-linolenic acid and seeds and stems exhibiting higher linoleic acid content [19]. Leaf extracts have been reported to contain substantial amounts of chlorophyll (≈305 mg/100 g) and carotenoids (≈69 mg/100 g). Moreover, mature leaves contain significantly higher concentrations of key carotenoids, including lutein isomers, violaxanthin, and β-carotene, compared with young leaves, further enhancing their nutritional and antioxidant value [19].

A unique hallmark of the species is the presence of *U. dioica* agglutinin (UDA), a chitin-binding antiviral lectin capable of inhibiting viral adsorption and entry by binding to viral glycoproteins. This bioactive compound underlies nettle’s antiviral potential and distinguishes it from many other medicinal plants. Regarding mineral composition, Table 1 presents the comprehensive mineral profile of *U. dioica*, including major macro-minerals (Ca, Mg, K, Na, P), essential micro-minerals (Fe, Mn, Sr, Ba, Cu, Zn, Ni, Cr, Ce, Co, La), and potentially toxic trace elements detected in smaller quantities. Calcium (16,500–24,890 mg/kg dw) and potassium (25,340–29,210 mg/kg dw) were the most abundant macro-minerals, while iron (267–442 mg/kg dw) represented the dominant micro-mineral [20,21,22].

Trace elements such as nickel, barium, chromium, cerium, and lanthanum were also detected. These diverse compounds have been recorded and characterized using integrated analytical techniques, including GC–MS for volatile compounds and fatty acids, FTIR for functional group identification, NMR and LC–MS/MS for structural elucidation, and HPLC/UPLC for phenolic profiling. These approaches provide a detailed chemical fingerprint of the species, which correlates strongly with its pharmacological functions and supports its application in nutraceutical, medicinal, and cosmetic products [23,24,25]. Table 2 presents the principal phytochemical groups identified in *U. dioica*.

**Figure 2:**
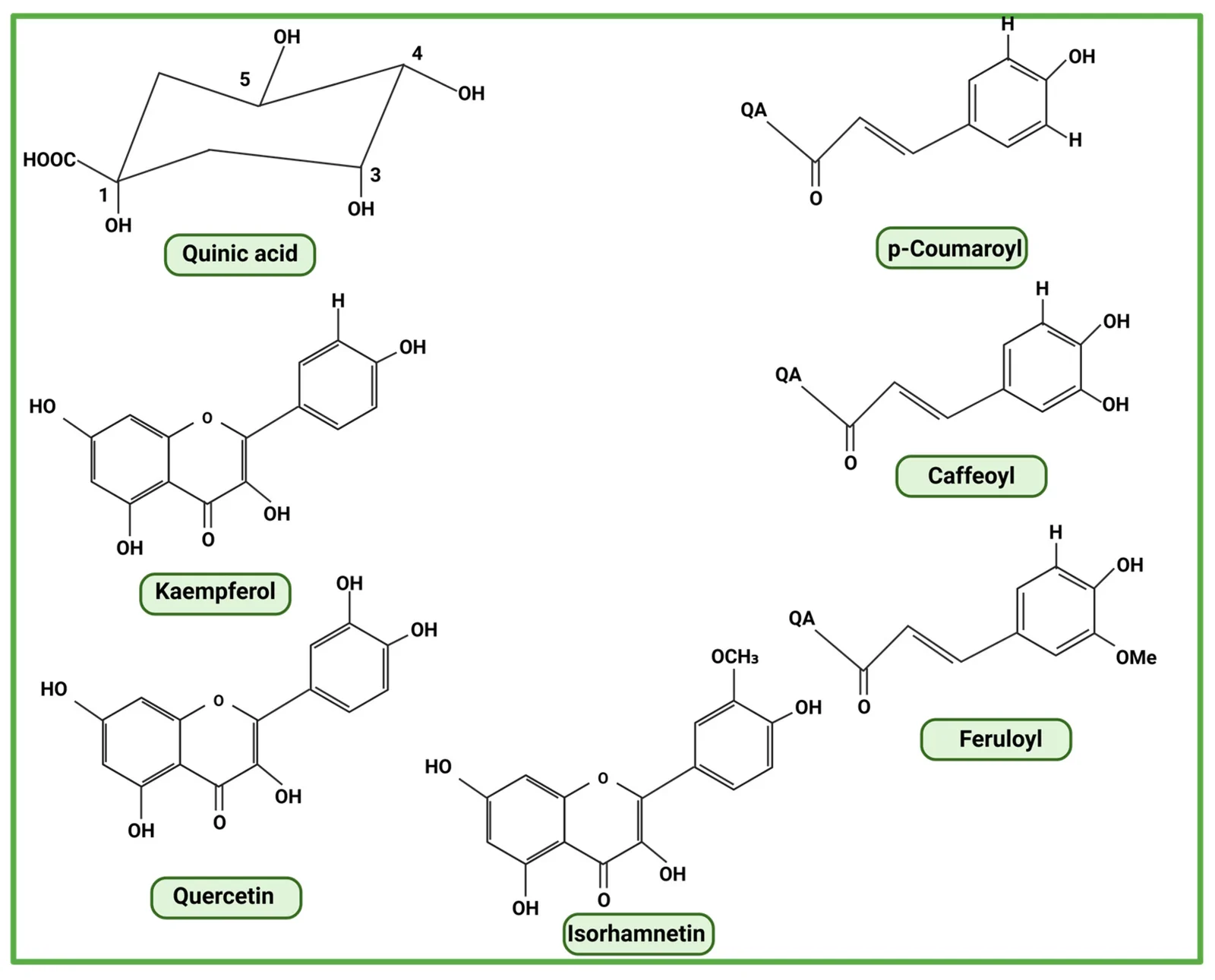
Major classes of natural products; phenolic acids, flavonoids, and fatty acids detected in *Urtica* Created in BioRender. Awlqadr, F. (accessed on 14 July 2026), https://BioRender.com/o91nj35.

Figure 2 illustrates the following compounds: hydroxycinnamic acids esterified with quinic acid, including *p*-coumaroyl, caffeoyl, and feruloyl derivatives; key flavonols and their glycosides, such as kaempferol, quercetin, and isorhamnetin; and a representative oxylipin (9-hydroxy-10,12-octadecadienoic acid) derived from polyunsaturated fatty acids.

## 5. Extraction Techniques

As shown in Table 3, extraction performance varies widely among conventional and green technologies, with methods such as UAE, MAE, PLE, and DES systems yielding higher phenolic recovery compared to traditional techniques.

### 5.1. Conventional Extraction Methods

Conventional extraction methods remain the cornerstone of phytochemical research on *U. dioica*. They rely on solvent diffusion and thermal processes to separate soluble bioactive compounds, such as polyphenols, flavonoids, vitamins, and essential oils, from plant tissues [31]. These methods (i.e., maceration, Soxhlet extraction, and hydrodistillation) provide reproducible results and are particularly suitable for isolating compounds such as chlorogenic acid, rutin, isorhamnetin derivatives, lignans, triterpenoids, and fatty acids [32]. Solvent selection critically affects both yield and bioactivity. According to Mitrović et al. [26], methanol–water (70%) extracts yielded the highest antioxidant activity (ABTS ≈ 94%) and total phenolic content (TPC ≈ 82 mg GAE/g) compared with ethanol or other pure solvents. Similarly, Naqash et al. [27] showed that adjusting solvent polarity improved polyphenol recovery from *U. dioica*, enhancing FRAP and DPPH activity by approximately 30–40%. Traditional extraction conditions typically involve temperatures of 25–60 °C for maceration (3–6 h) and up to 70 °C for Soxhlet reflux (up to 8 h), with solvent-to-sample ratios between 1:10 and 1:20 (*w*/*v*). Despite longer extraction times and higher solvent consumption, conventional methods remain widely used due to their simplicity and scalability.

#### 5.1.1. Maceration

Maceration is the simplest and most commonly used extraction technique for *U. dioica*. It involves soaking powdered plant material in a suitable solvent (commonly methanol, ethanol, hexane, or water) at moderate or slightly elevated temperatures. The extraction process depends on osmotic gradient formation and passive diffusion, which facilitates the transfer of soluble constituents over extended periods (8–72 h). Muceniece et al. [33] compared maceration for 6 h at 25 °C with sonication for *U. dioica* leaves and reported that maceration produced extracts with high antioxidant capacity but lower total polyphenol content (≈50 mg GAE/g) compared with sonication (≈68 mg GAE/g). Турдиева et al. [34] achieved 86.2% phenolic-acid and 95.4% flavonoid recovery using fivefold maceration for 6 h with 70% ethanol at room temperature per cycle, followed by liquid–liquid purification and spray drying at 175 °C inlet and 80 °C outlet temperatures to produce a stable dry extract. In another study, Naqash et al. [27] observed optimal polyphenol extraction using 60% methanol at 50 °C for 30 min, producing superior FRAP (0.82 μmol Fe^2+^/g) and DPPH (69.4%) activity. Similarly, Naderi et al. [35] found that aqueous maceration extracts (0.5 mg/100 mL) reduced malondialdehyde (MDA) formation and preserved sperm viability at 5 °C during 36 h of storage, demonstrating retention of bioactivity even under low-temperature extraction conditions. Key bioactive compounds recovered through maceration include chlorogenic acid, caffeic acid, rutin, lignans, quercetin glycosides, carotenoids, and β-sitosterol. These compounds contribute to the extract’s anti-inflammatory, antioxidant, and antimicrobial potential. Parente et al. [36] and Wójcik-Borowska et al. [25] linked macerated extracts of *U. dioica* with cytokine modulation (↑ IL-10, ↓ IL-1β, IL-6) and increased activity of cellular antioxidant enzymes (CAT, SOD). Advantages of this method include minimal thermal degradation and low equipment cost; however, its drawbacks include higher solvent consumption and longer extraction times. Nevertheless, maceration remains suitable for temperature-sensitive bioactive compounds in pharmaceutical and nutraceutical applications.

#### 5.1.2. Soxhlet Extraction

Soxhlet extraction provides continuous circulation of solvent through plant material, ensuring exhaustive extraction under controlled thermal conditions. Typically, 5–10 g of *U. dioica* powder is extracted using approximately 250 mL of methanol, ethanol, or hexane for 6–8 h at 60–80 °C. Mestour et al. [28] compared maceration and Soxhlet extraction for the recovery of essential oil from Algerian nettle and reported significantly higher yields using Soxhlet extraction (22.2–41.7%) compared with maceration (1.6–4.5%), highlighting Soxhlet’s efficiency in extracting lipophilic antioxidants and volatile terpenoids. Mitrović et al. [26] also reported the highest antioxidant activity and phenolic concentrations in methanol–water Soxhlet extracts among tested solvents (R^2^ > 0.95 correlation between DPPH and TPC). Naqash et al. [27] confirmed the suitability of Soxhlet extraction for isolating anthraquinones and phenolic acids from *U. dioica*, with HPLC analysis detecting caffeic acid, rutin, ferulic acid, quercetin, and kaempferol. Extraction times of 4–6 h at temperatures of 70–78 °C produced high-yield and stable extracts without significant degradation of thermostable compounds. The Soxhlet process offers advantages such as quantitative recovery and high reproducibility, making it suitable for analytical standardization of nettle-based formulations. However, high temperatures may degrade heat-labile compounds such as vitamin C, B-complex vitamins, and chlorophyll. In comparative analyses, Dirr and Karslioglu [37] reported decreased chlorophyll content after high-temperature Soxhlet treatment but also observed improved antioxidant capacity of essential-oil extracts. Extracts obtained using this method demonstrated strong anti-inflammatory and antibacterial activities due to enrichment in sterols, fatty acids, and phenolic compounds. For example, Parente et al. [36] associated Soxhlet ethanol extracts with inhibition of NF-κB activation and downregulation of COX-2 expression in inflammatory models. Thus, Soxhlet extraction remains a reference method for producing standardized bioactive extracts of *U. dioica* for food and pharmacological applications.

**Figure 3:**
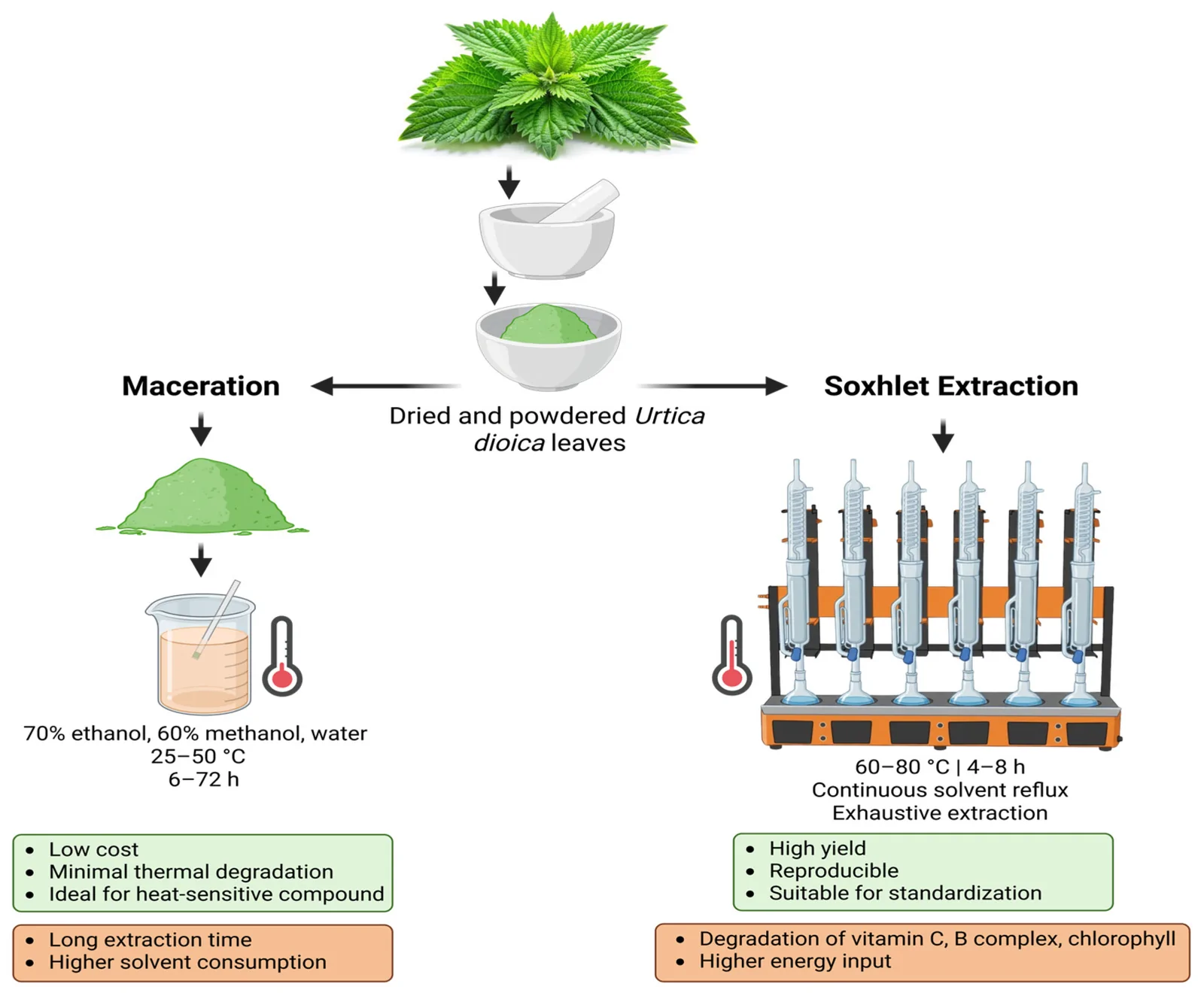
**Left**: Conventional maceration of *Urtica dioica* leaves low cost and gentle conditions but longer extraction time and higher solvent use. **Right**: Modern extraction methods higher yield and reproducibility, suitable for standardization, but with higher energy input and possible degradation of heat-sensitive compounds. Created in BioRender. Awlqadr, F. (14 July 2026), https://BioRender.com/gd731ia.

As illustrated in Figure 3, conventional maceration (left) provides a mild and low-cost extraction approach with minimal thermal degradation, whereas modern extraction techniques (right) offer higher yields and improved reproducibility but may require greater energy input and can compromise heat-sensitive compounds.

#### 5.1.3. Hydrodistillation

Hydrodistillation is considered a traditional method for the isolation of volatile constituents and essential oils from *U. dioica* stems and leaves using steam or water. The process involves immersing 50–100 g of dried plant material in 500–1000 mL of water and boiling it at approximately 95–100 °C for 2–3 h in a Clevenger-type apparatus, allowing for the condensation and collection of volatile oils. Mestour et al. [28] identified hydrodistillation as a key method for the characterization of nettle essential oils and compared its yields with Soxhlet and supercritical CO_2_ extraction. Hydrodistillation yields were relatively low (0.2–0.5%); however, the method preserves volatile esters and thermolabile terpenes responsible for antimicrobial and antioxidant properties. Common compounds recovered include β-caryophyllene, α-terpineol, carvacrol, hexadecanoic acid, and 1-octen-3-ol. These compounds contribute to broad-spectrum anti-inflammatory and antimicrobial activities. Nafeh et al. [7] demonstrated that infusion-derived extracts of *U. dioica* rich in volatile compounds enhanced cisplatin-induced apoptosis in SKOV-3 ovarian cancer cells via upregulation of caspase-3 and caspase-8. Similarly, Janicka et al. [38] reported strong virucidal activity of cold-brewed aqueous distillates against norovirus, indicating preservation of bioactive volatiles even under mild extraction conditions. Hydrodistillation temperature (≈100 °C) and extraction time (2–4 h) strongly affect both composition and yield. Excessive heating may cause oxidation or hydrolysis of terpenoids, while insufficient extraction time results in incomplete recovery. Optimal conditions for nettle essential oil extraction are typically around 3 h at atmospheric pressure, followed by solvent-free drying and GC-MS characterization. Extracted oils show notable antioxidant capacity (DPPH > 70%), enzyme-inhibitory effects, and potential skin-protective activities, as highlighted by Wójcik-Borowska et al. [25]. Hydrodistillation therefore remains indispensable for volatile profile characterization and for preparing essential-oil fractions used in cosmetics, pharmaceuticals, and food flavoring.

### 5.2. Modern Extraction Methods

#### 5.2.1. Ultrasound-Assisted Extraction

Ultrasound-assisted extraction (UAE) is a rapid and energy-efficient method that uses acoustic cavitation to disrupt plant cell walls, enhancing solvent penetration and the release of intracellular phytochemicals. For *U. dioica*, UAE has been widely optimized to recover phenolics, flavonoids, vitamins, and lignans. Naqash et al. [27] compared maceration, Soxhlet extraction, and UAE for polyphenol extraction and found that UAE using 60% methanol (1:15 *w*/*v*) at 40 °C for 25 min produced the highest total phenolic content (132.7 mg GAE/g) and flavonoid content (63.6 mg RE/g), along with the strongest antioxidant activities (DPPH ≈ 69%; ABTS ≈ 94%; FRAP ≈ 0.82 µmol Fe^2+^/g). Đurović et al. [39] further confirmed that UAE enhanced the recovery of polyphenols and vitamins B and C while maintaining thermal stability up to 160 °C, producing extracts rich in quercetin, rutin, chlorogenic acid, and caffeic acid. Typical UAE conditions for nettle extraction range between 30–50 °C, 15–30 min, and 100–400 W sonication power. Solvent choice strongly influences extraction selectivity: aqueous ethanol (50–70%) extracts higher amounts of flavonoids, whereas water favors the extraction of phenolic acids and vitamins. Sonication improves antioxidant enzyme activation and antimicrobial capacity by increasing the dissolution of hydrophilic antioxidants. Muceniece et al. [33] reported that UAE-treated aqueous extracts improved hepatoprotective activity by reducing lipid accumulation in HepG2 cells through enhanced mitochondrial oxygen consumption.

#### 5.2.2. Microwave-Assisted Extraction

**Figure 4:**
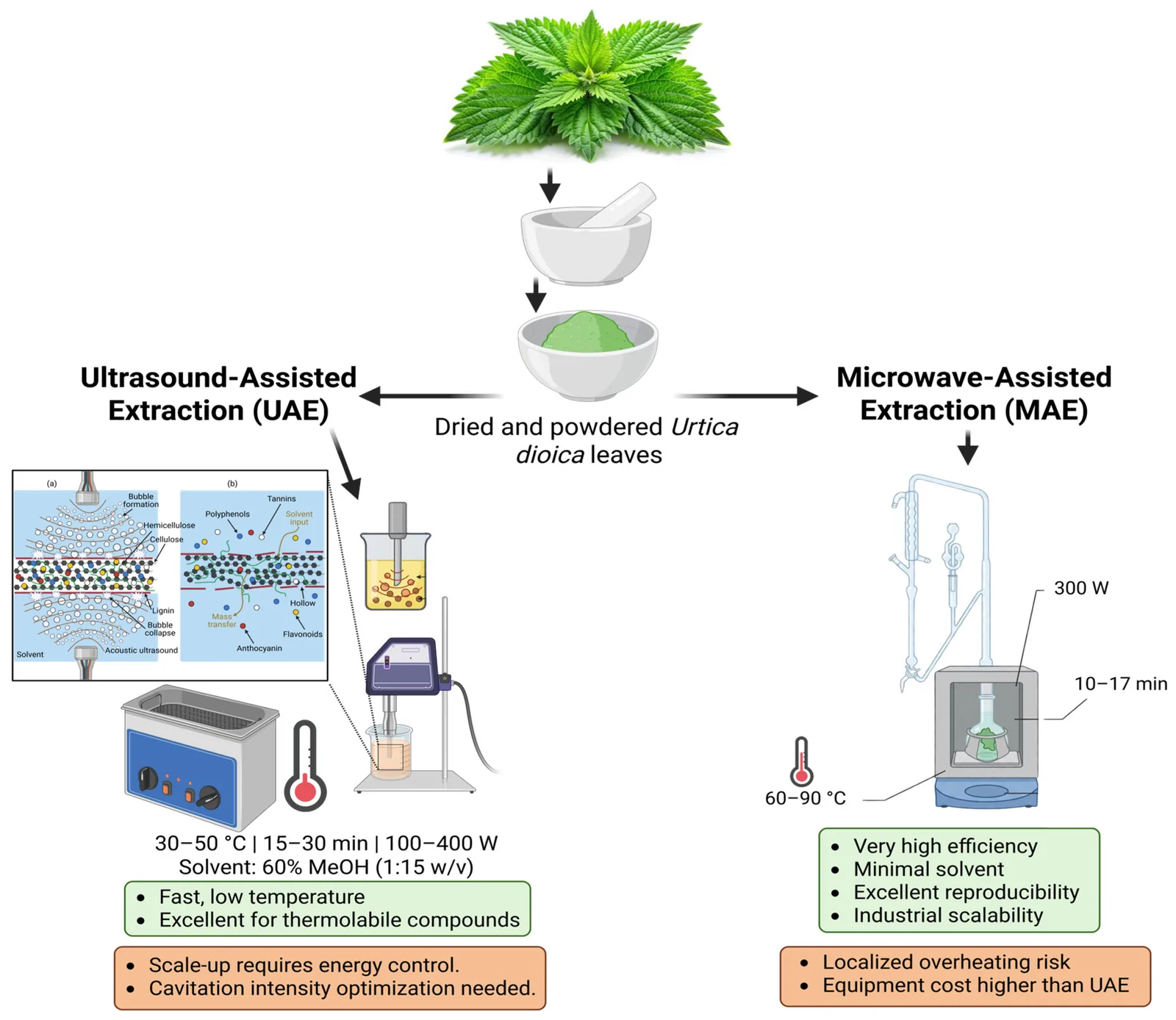
Schematic comparison of green extraction methods applied to for *Urtica dioica* leaves for the recovery of bioactive compounds: (**left**) ultrasound-assisted extraction (rapid, performed at low temperature, and suitable for thermolabile compounds); (**right**) microwave-assisted extraction (highly efficient, reproducible, and industrially scalable). The inset illustrates acoustic cavitation during UAE: (**a**) the plant cell wall during ultrasonic treatment and (**b**) cell-wall disruption after UAE, facilitating mass transfer and the release of bioactive compounds. Created in BioRender. Awlqadr, F. (14 July 2026), https://BioRender.com/qh80jlt.

Microwave-assisted extraction (MAE) employs dielectric heating to rapidly disrupt plant tissues, releasing bound metabolites within minutes. Sahal et al. [15] optimized *U. dioica* MAE using response surface methodology (RSM) with extraction times of 10–17 min at 300 W and solvent-to-sample ratios of 1:10–1:13 (*w*/*v*) using 80% ethanol, water, and natural deep eutectic solvent (NADES; Choline chloride (ChCl): lactic acid). The NADES-MAE system produced the highest total phenolic content (≈108 mg GAE/g), radical scavenging activity (DPPH ≈ 93%), and total flavonoid content (≈46 mg QE/g). MAE offers several advantages, including minimal solvent consumption, high extraction yield, and strong reproducibility. Extraction temperatures typically range from 60–90 °C depending on microwave power and solvent polarity. Naseem et al. [40] further expanded MAE applications to cellulose recovery using a Sono-Microwave Assisted Chlorine-free Ionic Liquid (SMACIL) system (BMIM-Ac ionic liquid + H_2_O_2_, 90 °C, 15 min), achieving 88% cellulose yield with improved tensile strength and crystallinity. MAE-derived nettle extracts display multiple bioactivities, including antimicrobial activity against *S. aureus* and *E. coli*, reactive oxygen species (ROS) scavenging, and mitochondrial protection. As shown in Figure 4, powdered *U. dioica* leaves were extracted using ultrasound-assisted extraction (left), which is rapid and suitable for thermolabile compounds, and microwave-assisted extraction (right), which provides very high efficiency and reproducibility with industrial scalability but carries a risk of localized overheating.

#### 5.2.3. Supercritical Fluid Extraction

Supercritical CO_2_ extraction (SFE) employs carbon dioxide above its critical point (31 °C, 74 bar) to recover non-polar and moderately polar compounds without thermal degradation [41]. Mestour et al. [28] evaluated SFE of *U. dioica* leaves and stems at 250 bar and 40 °C, obtaining essential-oil yields of 0.50% and 0.21%, respectively—lower than Soxhlet extraction but chemically richer in α-terpineol, β-caryophyllene, and hexadecanoic acid. The extracts displayed high antioxidant and anti-inflammatory activity, making them suitable for pharmaceutical and cosmetic applications. Typical SFE parameters for nettle bioactives include pressures of 200–350 bar, temperatures of 40–60 °C, extraction times of 60–120 min, and CO_2_ flow rates of approximately 2–4 L min^−1^, sometimes with ethanol used as a co-solvent (5–10%). Đurović et al. [39] noted that SFE preserved heat-sensitive vitamins B and C while enriching triterpenoids and carotenoids, thereby enhancing antioxidant stability. Naderi et al. [35] further confirmed that lipid fractions obtained under supercritical conditions improved antioxidant defense in biological systems by reducing malondialdehyde levels in stored semen samples due to retained fatty acids and sterols. Supercritical extracts of *U. dioica* are particularly rich in sterols (β-sitosterol, stigmasterol), terpenoids (β-amyrin, lupeol), and unsaturated fatty acids, which exhibit strong anti-inflammatory and cardioprotective effects.

#### 5.2.4. Pressurized Liquid Extraction

**Figure 5:**
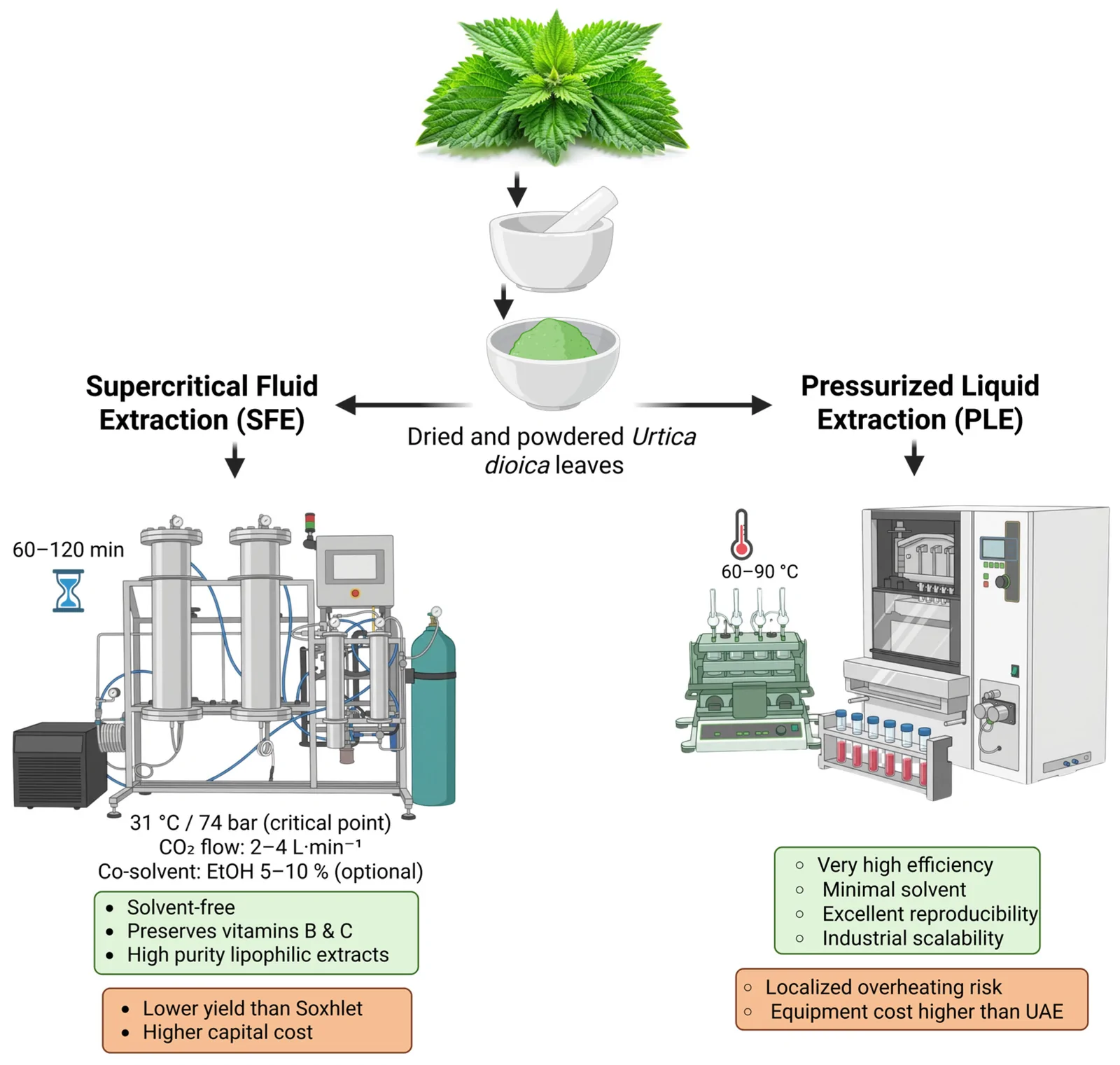
Green extraction of *Urtica dioica* leaves: **left**, supercritical CO_2_ extraction (solvent-free, high-purity extracts); **right**, pressurized liquid extraction (fast, reproducible, industrially scalable). Created in BioRender. Awlqadr, F. (23 July 2026), https://BioRender.com/d2z72ok.

Pressurized liquid extraction (PLE), also known as accelerated solvent extraction, uses elevated temperatures (50–200 °C) and pressures (10–15 MPa) to maintain solvents in the liquid state, thereby improving mass transfer and extraction kinetics. Cegledi et al. [42] optimized PLE for *U. dioica* roots, targeting lipid fractions rich in phytosterols and pentacyclic triterpenoids. Optimal conditions (150 °C, 5 min × 4 cycles, ethanol) yielded a 1.63% extract containing β-sitosterol (50.2 mg/100 g) and β-amyrin acetate (0.56 mg/100 g), with concentrations approximately fourfold higher than those obtained using Soxhlet extraction (68 °C, 8 h). PLE typically operates between 100 and 200 °C for 5–20 min per cycle, allowing for the extraction of both polar and nonpolar compounds depending on solvent selection. Compared with Soxhlet extraction, PLE significantly reduces extraction time and solvent consumption by up to 90%, while minimizing compound degradation. It efficiently recovers phytosterols, triterpenoids, and phenolic antioxidants, which are beneficial for lipid metabolism, anti-aging, and anti-inflammatory functions. Moreover, its closed-system operation ensures reproducibility and suitability for large-scale industrial applications. When combined with GC-MS and UPLC-DAD-MS profiling, PLE enables the production of chemically well-defined nettle extracts of high purity for use in dietary supplements, functional foods, and cosmeceuticals. This finding is further supported by Repajić et al. [30], who optimized PLE for polyphenols and pigments recovery from *U. dioica* leaves and showed that increasing the extraction temperature to 110 °C for 10 min significantly enhanced total phenolic content and antioxidant capacity, producing an extract with 60% higher antioxidant capacity. As illustrated in Figure 5, supercritical CO_2_ extraction (left) enables solvent-free recovery of high-purity lipophilic compounds from *U. dioica*, whereas pressurized liquid extraction (right) provides a rapid, reproducible, and industrially scalable alternative.

### 5.3. Optimization Strategies for Extraction Yield

Optimization of extraction yield in *U. dioica* relies on adjusting multiple operational parameters—temperature, solvent polarity, extraction time, and solid-to-solvent ratio—using statistical modeling and advanced experimental design tools. According to Naqash et al. [27], response surface methodology (RSM) effectively maximized total flavonoid and phenolic yields by optimizing methanol concentration (60%), extraction time (30 min), and temperature (50 °C), resulting in DPPH activity of 69.4% and total phenolic content (TPC) of 132.7 mg GAE/g. Similarly, Sahal et al. [15] optimized microwave-assisted extraction (MAE) using a Box–Behnken model with extraction time (10–20 min), microwave power (300–600 W), and solvent ratio (1:10–1:20, *w*/*v*). Under these conditions, the use of a NADES system composed of choline chloride and lactic acid produced 108 mg GAE/g TPC and antioxidant activity exceeding 93% in the DPPH assay.

Optimization approaches integrate desirability-function modeling with response surface design, enabling prediction of ideal conditions for multiple response variables. Koraqi et al. [29] reported that optimization of DES–UAE parameters (20% water addition, 70 °C, 30 min) using central composite design resulted in exceptionally high yields, with total flavonoid content (TFC) of 134.7 mg CE/100 g and TPC of 2423 mg GAE/100 g. Furthermore, solvent polarity plays a decisive role: ethanol–water mixtures (60–80%) efficiently extract both lipophilic and hydrophilic phenolics, whereas DES systems improve the selectivity and bioavailability of compounds such as chlorogenic acid, quercetin, and caffeoylmalic acid. Garofulić et al. [43] demonstrated that pressurized liquid extraction (PLE) combined with ethanol (15 MPa, 150 °C) produced the highest ORAC antioxidant capacity and total phenolic content, outperforming conventional heat-reflux extraction. Therefore, combining empirical modeling, innovative solvent systems, and kinetic analysis ensures maximal recovery of bioactive compounds while maintaining their chemical integrity. These optimized extraction strategies provide a scientific foundation for reproducible, scalable, and sustainable production of nettle-derived nutraceuticals.

### 5.4. Sustainability and Green Extraction Approaches

Sustainability in *U. dioica* extraction emphasizes the transition from energy-intensive and solvent-consuming conventional processes toward environmentally friendly green extraction technologies. Conventional methods such as Soxhlet extraction and maceration often require long processing times (8–72 h) and the use of volatile organic solvents (e.g., hexane or methanol), resulting in environmental waste and high energy consumption. In contrast, modern green extraction techniques—including microwave-assisted extraction (MAE), ultrasound-assisted extraction (UAE), supercritical fluid extraction (SFE), and deep eutectic solvent (DES) systems—offer higher extraction efficiency with reduced environmental impact [44].

Koraqi et al. [29] pioneered the use of food-grade deep eutectic solvents composed of citric acid and maltose (DESCAMAL) combined with UAE, achieving exceptionally high extraction yields (DPPH 1071 µmol TE/mL; TPC 2423 mg GAE/100 g) under mild conditions (30 min, 70 °C) using GRAS-certified biodegradable components. These NADES systems function as tunable green solvents with strong solvation capacity for flavonoids and phenolic compounds and can be recycled without leaving toxic residues. Similarly, Kowalska et al. [45] introduced water–glycerol mixtures (87.5:12.5%) as an alternative solvent system, optimizing extraction at temperatures between 20 and 50 °C and achieving high recovery of chlorophyll and polyphenols with strong environmental compatibility.

From an energy-consumption perspective, MAE and UAE significantly reduce extraction time to less than 30 min and decrease energy input by enhancing cell wall disruption through dielectric heating and acoustic cavitation. Supercritical fluid extraction using CO_2_ operates at relatively low temperatures (40–50 °C) and eliminates the need for organic solvents, producing lipid-rich extracts containing carotenoids, β-sitosterol, and terpenes. Pressurized liquid extraction (PLE), optimized by Garofulić et al. [42], employs water or ethanol under pressure to efficiently recover phenolic compounds while reducing solvent consumption by more than 90%.

Beyond methodological efficiency, sustainability also includes biorefinery integration, where multiple plant parts (leaves, stems, and roots) are valorized to minimize waste. Extraction residues may be repurposed as animal feed or organic fertilizer, supporting a circular-economy framework. Emerging techniques such as pulsed electric field (PEF) and enzyme-assisted extraction (EAE) further enhance mass transfer through cell membrane permeabilization, reducing the need for solvents and high temperatures. Garofulić et al. [43] suggested that hybrid systems combining PLE or MAE with NADES could represent the next generation of “zero-waste” bioprocessing for herbal materials.

Thus, the transition toward green chemistry principles—including renewable solvents, reduced energy consumption, minimal waste generation, and safe residues—defines the modern paradigm of sustainable extraction. By integrating optimization tools (CCD, RSM), eco-friendly solvents (ethanol–water mixtures, DES, glycerol), and clean technologies (MAE, UAE, SFE), researchers can achieve high extraction yields with a low environmental footprint while preserving sensitive phytochemicals. These advancements not only strengthen the nutraceutical and cosmetic potential of *U. dioica* but also align with the United Nations Sustainable Development Goals (SDGs 12 and 13) for responsible production and climate action.

## 6. Characterization of Bioactive Compounds

### 6.1. Phytochemical Screening Methods

Phytochemical screening provides qualitative evidence of the diverse secondary metabolites present in *U. dioica*, including flavonoids, lignans, phenolic acids, sterols, terpenoids, and alkaloids. Routine screening methods include colorimetric assays (Folin–Ciocalteu, vanillin–H_2_SO_4_, and AlCl_3_), thin-layer chromatography, and UV–Vis spectroscopy for the detection of flavonoids and total phenolics. Devkota et al. [46] identified rutin, kaempferol, and chlorogenic acid as the principal phenolic compounds correlated with antioxidant potential. Tarasevičienė et al. [47] optimized phenolic extraction (70 °C, 96% methanol) and demonstrated that leaves contain higher levels of polyunsaturated fatty acids (PUFAs) and carotenoids than roots. Similarly, Engelhardt et al. [48] quantified changes in polyphenol content following drying and cooking and observed increased antioxidant activity due to cell-wall disruption.

Preliminary phytochemical screening revealed high total phenolic content (≈47 mg GAE g^−1^) and flavonoid content (≈17 mg QE g^−1^) in methanolic extracts of nettle [49]. Niaz et al. [50] reported the presence of tannins, saponins, and alkaloids in both roots and leaves, confirming the plant’s broad pharmacological potential. Extraction conditions significantly influence phytochemical outcomes: moderate heating (60–70 °C) enhances diffusion and extraction efficiency, whereas excessive heating (>90 °C) may lead to phenolic oxidation. Phytochemical fingerprints also guide the selection of downstream chromatographic or spectroscopic techniques for compound quantification and structural elucidation. Overall, qualitative phytochemical profiling establishes the biochemical basis for nettle’s antioxidant, antidiabetic, and anti-inflammatory activities [39,49,50].

### 6.2. Chromatographic Techniques

Chromatographic techniques enable the separation and precise quantification of nettle bioactive compounds across a wide polarity range. High-performance liquid chromatography (HPLC) is considered the gold standard for identifying phenolic acids, flavonoids, and vitamins [51]. The ability of chromatographic techniques to resolve compounds across a wide polarity range depends on differential partitioning between the stationary and mobile phases. In reversed-phase UPLC/HPLC, the nonpolar C18 stationary phase retains hydrophobic analytes, such as flavonoid aglycones, through van der Waals or hydrophobic interactions, while more polar compounds, such as chlorogenic acid derivatives and phenolic acids, elute earlier because of their stronger affinity for the polar aqueous mobile phase and weaker retention on the nonpolar stationary phase. DAD/UV detection exploits the UV absorbance characteristic of aromatic chromophores, such as absorbance at 350 nm for flavonoids and 280 nm for phenolic compounds), enabling both quantification and identification based on Beer–Lambert law-governed peak area-concentration relationships. Collectively, these complementary separation mechanisms, including hydrophobic partitioning and UV/MS detection in LC-based techniques, together with volatility-driven partitioning and EI-MS fragmentation in GC-MS allow chromatographic methods to comprehensively resolve and quantify nettle bioactives, ranging from highly polar phenolic acids to non-polar volatile terpenes and fatty acids [29,51]. Engelhardt et al. [48] applied HPLC-DAD (C18 column, 25 °C, 1 mL/min, 40 min gradient) to quantify 5-caffeoylquinic acid, rutin, and quercetin. Tarasevičienė et al. [47] extracted phenolics using 96% methanol at 70 °C and quantified them by HPLC-UV (280 nm), reporting higher yields in leaves. Devkota et al. [46] coupled HPLC with diode-array detection to characterize carotenoids (69 mg/100 g) and chlorophylls (305 mg/100 g). The analytical precision of HPLC depends on mobile-phase composition (methanol or acetonitrile with acidified water, pH 2.8–3.2) and extraction temperature (50–70 °C).

Ultra-performance liquid chromatography (UPLC) improves throughput and resolution through the use of sub-2 µm particle columns. Jeszka-Skowron et al. [52] used UPLC-MS to quantify phenolic acids (caffeic, ferulic, and coumaric acids) and trigonelline across different plant parts, demonstrating variations between roots and leaves. UPLC reduces run time (<10 min) and solvent consumption by approximately 40% compared with conventional HPLC. However, instrumentation costs are higher, and columns are more fragile.

Gas chromatography–mass spectrometry (GC–MS) is particularly suitable for analyzing volatile and semivolatile compounds. Mihaylova et al. [49] profiled 48 volatile constituents, including linalool, hexadecanoic acid, and β-caryophyllene, using GC–MS (HP-5 MS column, 60–250 °C temperature gradient, helium flow rate 1 mL/min). Đurović et al. [39] applied GC–MS following supercritical CO_2_ extraction to identify α-linolenic, palmitic, and linoleic acids. Retention time alignment and mass spectral library comparisons (NIST and Wiley) were used to confirm compound identity. GC–MS complements LC-based techniques by revealing essential oil and lipid constituents responsible for antimicrobial and cardioprotective effects. Table 4 summarizes the chromatographic techniques used for profiling *U. dioica*, outlining the analyte types, operating conditions, advantages, and limitations of HPLC–DAD, UPLC–MS, and GC–MS.

### 6.3. Spectroscopic Techniques

Spectroscopic analyses are widely used to elucidate molecular structures and confirm the purity of *U. dioica* extracts. Fourier-transform infrared spectroscopy (FTIR) identifies functional groups responsible for antioxidant activity. Tarasevičienė et al. [47] reported characteristic absorption peaks at 3400 cm^−1^ (–OH), 1650 cm^−1^ (C=O), and 1045 cm^−1^ (C–O–C), indicating the presence of phenolic hydroxyl groups and glycosidic linkages. Đurović et al. [39] used FTIR to confirm ester C=O and C–H stretching vibrations in lipid extracts. FTIR requires minimal sample preparation and complements chromatographic techniques used for compound quantification.

Nuclear magnetic resonance (NMR) spectroscopy provides definitive structural elucidation of nettle metabolites. Jeszka-Skowron et al. [52] employed ^1^H NMR (CD_3_OD, 400 MHz) to confirm characteristic peaks of nicotinamide and trigonelline at δ 8.6–9.0 ppm. Devkota et al. [46] also highlighted the role of NMR in the characterization of fatty acids and sterols. Two-dimensional NMR techniques (e.g., HSQC and COSY) allow the resolution of overlapping aromatic signals in flavonoid mixtures, although they require larger sample quantities (≥10 mg).

Liquid chromatography–tandem mass spectrometry (LC–MS/MS) combines chromatographic separation with structural fragmentation analysis. Jeszka-Skowron et al. [52] quantified 20 phenolic and nitrogen-containing compounds, detecting trigonelline at concentrations ranging from 2.8 to 108 µg g^−1^ across different plant organs. Đurović et al. [39] identified phenolic acids in microwave-extracted nettle-enriched bread, monitored at 312 nm absorbance. Characteristic MS/MS transitions (*m*/*z* 301 → 151 for quercetin; *m*/*z* 353 → 191 for chlorogenic acid) enable highly sensitive quantification with detection limits below 10^−9^ M.

These spectroscopic platforms therefore authenticate and quantify key compounds responsible for the biological efficacy of nettle, including rutin (antioxidant), quercetin (anti-inflammatory), β-sitosterol (cholesterol-lowering), and linolenic acid (cardioprotective). Table 5 provides an overview of the principal spectroscopic techniques applied in the analysis of *U. dioica*, detailing their purposes, operational parameters, advantages, and limitations for FTIR, NMR, and LC–MS/MS.

## 7. Health-Promoting Effects

### 7.1. Antioxidant Properties

**Figure 6:**
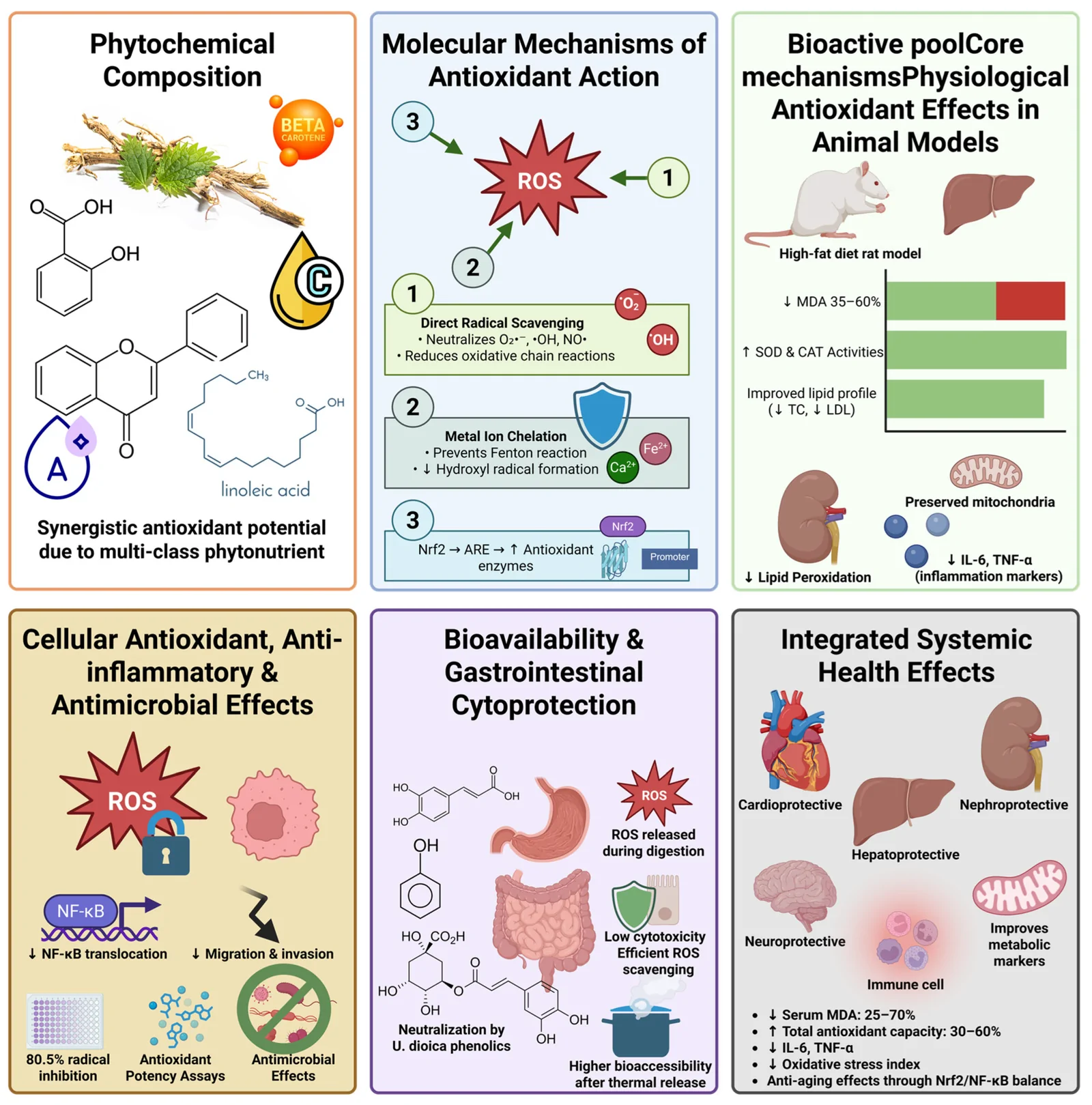
Overview of the proposed antioxidant, anti-inflammatory, antimicrobial, gastrointestinal, and systemic cytoprotective effects of *Urtica dioica*. Created in BioRender. Awlqadr, F. (14 July 2026), https://BioRender.com/os2ugz1.

The antioxidant system of *U. dioica* plays a pivotal role in protecting cellular macromolecules from oxidative injury, thereby preventing the onset or progression of metabolic, cardiovascular, hepatic, renal, and neurodegenerative disorders. Comprehensive compositional studies reveal high concentrations of polyphenols (rutin, kaempferol, quercetin, chlorogenic acid, and caffeic acid), vitamins A and C, carotenoids, and unsaturated fatty acids that synergistically modulate redox homeostasis [53]. Devkota et al. [46] emphasized that these constituents act through direct radical-scavenging and metal-chelating reactions, as well as by upregulating endogenous antioxidant enzymes such as superoxide dismutase (SOD), catalase (CAT), and glutathione peroxidase (GPx), thereby providing broad-spectrum cytoprotection. See Figure 6 for a visual summary of the antioxidant, anti-inflammatory, and cytoprotective mechanisms of *U. dioica*.

In vivo evidence supports these systemic benefits. In rats subjected to high-fat diets or chemically induced oxidative stress, *U. dioica* leaves extract and seeds significantly reduced hepatic lipid peroxidation malondialdehyde decreased by 35–60% and restored SOD and CAT activities toward normal levels [54,55,56]. These improvements coincided with better serum lipid profiles, indicating that antioxidant mechanisms translate into cardiometabolic protection. Clinical and observational data in individuals with mild hyperlipidemia similarly show that nettle supplementation decreases total and LDL cholesterol while increasing plasma antioxidant capacity, suggesting potential utility as a nutraceutical for metabolic syndrome management.

Cell culture studies extend these findings to cytoprotective and anti-inflammatory contexts. Dakhli et al. [24] demonstrated that aqueous nettle leaf extract rich in phenolic compounds inhibited reactive oxygen species (ROS) accumulation, suppressed NF-κB activation, and reduced the migratory and invasive behavior of human colon carcinoma (HCT-116) cells. Fayza et al. [57] reported strong free-radical scavenging activity (80.5% DPPH inhibition) and metal-chelating capacity in both leaf and root extracts, correlating with bactericidal activity against *E. coli* and *S. aureus*. These findings suggest that oxidative stress mitigation contributes to antimicrobial defense.

Nettle’s antioxidant potential also benefits reproductive and neural tissues. Engelhardt et al. [48] demonstrated that cooking and digestion release bound 5-caffeoylquinic acid, increasing antioxidant bioavailability and explaining why cooked nettle dishes retain high nutritional value. Furthermore, human intestinal Caco-2 cell assays confirmed low cytotoxicity and effective ROS scavenging, indicating safety and intestinal compatibility. The systemic integration of these effects leads to measurable reductions in biomarkers of oxidative stress and inflammation. Jaiswal and Lee [58] summarized more than 20 animal studies in which nettle administration reduced serum malondialdehyde levels by 25–70%, increased total antioxidant capacity by 30–60%, and attenuated inflammatory cytokines such as IL-6 and TNF-α. Such modulation of redox-sensitive signaling pathways, particularly activation of Nrf2 and inhibition of NF-κB, underlies the observed hepatoprotective, nephroprotective, and anti-aging effects. Collectively, the phytochemicals of *U. dioica* may act synergistically through direct ROS scavenging, chelation of pro-oxidant metals, and activation of the Nrf2–ARE signaling pathway. These mechanisms enhance endogenous antioxidant defenses, including SOD, CAT, and GPx activities, while limiting lipid peroxidation and preserving mitochondrial integrity. Its anti-inflammatory effects are primarily associated with suppression of NF-κB signaling and reduced production of pro-inflammatory mediators, including IL-6 and TNF-α. Following digestion, the intestinal availability and transformation of phenolic constituents may also support epithelial ROS scavenging and gastrointestinal cytoprotection. In addition, antimicrobial activity may reduce pathogen-related cellular injury. Preclinical studies have further reported improvements in oxidative stress biomarkers, lipid metabolism, inflammatory balance, and organ-specific protection; however, most of these systemic effects remain supported predominantly by in vitro and animal evidence.

### 7.2. Anti-Inflammatory Effects

Extensive experimental and preclinical evidence demonstrates that *U. dioica* exerts strong anti-inflammatory activity through multiple complementary mechanisms. Polyphenol-rich fractions of nettle leaves and flowers suppress lipopolysaccharide (LPS)- and hydrogen peroxide-induced inflammation in human skin fibroblasts by lowering interleukin (IL)-1β and IL-6 release, reducing intracellular reactive oxygen species (ROS), and upregulating superoxide dismutase (SOD) and catalase (CAT) activities, with chlorogenic acid and isorhamnetin derivatives identified as major contributors [25]. In particular, in chondrocytes stimulated with IL-1β, nettle extracts inhibited IκBα degradation and NF-κB activation, decreased cyclooxygenase-2 (COX-2) and matrix metalloproteinases (MMP-9 and MMP-13), and preserved collagen II, demonstrating chondroprotective and anti-catabolic effects [59].

Essential oil from nettle leaves (UDEO) has also shown potent anti-inflammatory activity in vivo. In the carrageenan-induced paw edema model, rats receiving UDEO exhibited significantly reduced edema volume and C-reactive protein (CRP) levels, together with increased SOD, CAT, and glutathione peroxidase (GPx) activities. β-Linalool, phytol, menthol, and 1,8-cineole were identified as the main anti-inflammatory constituents [60]. Similarly, methanolic root extracts attenuated acetic acid-induced colitis in rats, lowering myeloperoxidase (MPO) and nitric oxide (NO) levels while enhancing antioxidant enzyme activity [61]. In macrophage (RAW 264.7) cultures, 70% ethanolic leaf extract suppressed nitric oxide production (IC_50_ ≈ 95 µg/mL) and downregulated tumor necrosis factor-α (TNF-α) and IL-1β expression [2].

Clinical relevance is supported by ex vivo and systemic models. In human peripheral blood mononuclear cells, nettle infusion reduced TNF-α and IL-1β secretion by nearly half and increased intracellular glutathione levels [62]. Comparable hepatoprotective and anti-inflammatory effects were reported in carbon tetrachloride-treated rats, where aqueous–methanolic extracts lowered alanine aminotransferase (ALT) and aspartate aminotransferase (AST) levels, reduced TNF-α, and inhibited NF-κB nuclear translocation [2,63]. In diabetic animals, ethanolic nettle leaf extract decreased serum IL-6 and CRP levels and restored pancreatic β-cell integrity [64].

Finally, a recent comprehensive review confirmed consistent downregulation of NF-κB, COX-2, inducible nitric oxide synthase (iNOS), and pro-inflammatory cytokines across cellular and animal models [65]. Together, these findings establish *U. dioica* as a multifunctional botanical anti-inflammatory agent. Its phenolics, flavonoids, terpenoids, and sterols act synergistically to modulate oxidative stress, inhibit key inflammatory signaling pathways, and promote tissue repair mechanisms, supporting its expanding use in nutraceutical, dermatological, and joint-health formulations.

### 7.3. Antimicrobial and Antiviral Action

Recent advances in extraction technologies and molecular profiling have increased our understanding of the antimicrobial and antiviral properties of *U. dioica* L., a plant rich in phenolic acids, flavonoids, lectins, and other secondary metabolites. These bioactive compounds contribute to its broad-spectrum activity against bacterial, fungal, and viral pathogens, highlighting its importance in modern phytotherapy and pharmacological research.

Recent extraction technologies have played a critical role in enhancing the antimicrobial efficacy of *U. dioica*. Optimal results have been achieved using natural deep eutectic solvents (NADES) for the extraction of phenolic and flavonoid compounds, resulting in enhanced antibacterial activity against *Staphylococcus aureus*, *Streptococcus pyogenes*, and *Escherichia coli*. Under optimal microwave-assisted extraction (MAE) conditions (300 W, 17 min), extracts exhibited significantly higher total phenolic content (TPC) and total flavonoid content (TFC), leading to improved antimicrobial activity. Similarly, silver nanoparticles (AgNPs) synthesized using nettle extracts demonstrated strong antimicrobial properties and enhanced oxidative stability [66]. Mahmodabad et al. [67] reported that ultrasound-assisted AgNPs inhibited *E. coli*, *S. aureus*, *Candida albicans*, and *Aspergillus niger*, and improved food preservation by reducing total volatile nitrogen and peroxide levels by 50–70% during refrigerated storage of shrimp.

Hashem and Salem [68] reported complementary findings by synthesizing selenium nanoparticles (SeNPs) using *U. dioica* leaf extract through green synthesis methods. The resulting SeNPs showed strong antibacterial activity with minimum inhibitory concentrations (MICs) of 31.25–500 µg/mL against *E. coli*, *Bacillus subtilis*, and *S. aureus*, as well as antifungal activity against *Candida albicans* and *Aspergillus* species, while exhibiting low cytotoxicity (IC_50_ = 173.2 µg/mL) toward normal Vero cells. These nanoparticle systems indicate that antimicrobial effects may be synergistically mediated by metal ions and plant phytochemicals, leading to microbial membrane disruption and oxidative stress in pathogens.

Conventional solvent-based extracts of *U. dioica* also exhibit notable antibacterial activity. Koszegi et al. [69] found that ethanolic leaf extracts harvested in spring contained the highest polyphenol concentrations and showed strong antibacterial activity against *E. coli* and *S. aureus*, while aqueous extracts were most effective against the growth of *Candida glabrata*. Similarly, Gülhan and Yangilar [70] demonstrated bacteriostatic and bactericidal activity against six clinical pathogens, with the strongest inhibition observed against *Streptococcus pneumoniae* and *S. aureus*. Salehzadeh et al. [71] further confirmed the activity of nettle extracts against methicillin-resistant *S. aureus* (MRSA) isolates (MICs ≈ 20 mg/mL), suggesting their potential as natural antiseptic alternatives.

*U. dioica* has also demonstrated promising antiviral properties. Aydin et al. [72] reported that methanolic extracts inhibited the infectivity of murine norovirus (MNV-1) by approximately 50% at concentrations of 1.45–1.87 mg/mL and also inhibited the growth of *Campylobacter jejuni* (MIC = 5 mg/mL). Similarly, Janicka et al. [38] demonstrated near-complete inactivation of norovirus by cold-extracted nettle preparations, which correlated with strong antioxidant activity. In addition to extract-based antiviral effects, lectins such as *U. dioica* agglutinin (UDA) have been identified as potent antiviral proteins capable of binding glycosylated viral envelope proteins and inhibiting viral entry into host cells [4].

Overall, these antimicrobial and antiviral activities are primarily mediated through phenolic-driven redox regulation, microbial membrane destabilization, and lectin–glycoprotein interactions [57,73] (Table 6).

Taken together, the available evidence indicates that *U. dioica* represents a sustainable and multifunctional source of natural antimicrobial and antiviral agents, confirming its traditional therapeutic value while demonstrating its compatibility with modern extraction technologies and nanotechnology-based systems.

### 7.4. Antidiabetic Effects

*U. dioica*, commonly known as stinging nettle, has been extensively investigated for its antidiabetic properties through in vitro experiments, preclinical models, and clinical studies conducted over the past two decades. In various experimental models, *U. dioica* exhibits significant hypoglycemic effects through multiple mechanisms, including stimulation of insulin secretion, regulation of glucose uptake, inhibition of α-glucosidase and α-amylase enzymes, and improvement of lipid metabolism and oxidative stress parameters.

An insulin-secretagogue fraction (F-1) isolated from *U. dioica* leaves by Farzami et al. [75] significantly increased insulin secretion from isolated pancreatic Langerhans islets and reduced blood glucose levels in streptozotocin (STZ)-induced diabetic rats. This effect was directly stimulated by the insulin-secretagogue fraction (F-1) at concentrations ranging from 0.05 to 3.0 M. Similarly, Bnouham et al. [76] verified the antihyperglycemic activity of an aqueous extract in oral glucose tolerance tests (OGTT), demonstrating a 33% reduction in glycemia through inhibition of intestinal glucose absorption. Molecular characterization by Rehman et al. [77] identified caffeoylmalic acid as one of the major bioactive constituents responsible for strong α-amylase and α-glucosidase inhibitory activity and stimulation of glucose uptake in in vitro models. This compound also reduced blood glucose levels as well as lipid and hepatic biomarkers in diabetic mice.

Mehran et al. [78] compared the effects of *U. dioica* and *Lamium album* extracts (100 mg/kg/day for 28 days) in STZ-induced diabetic rats and reported that the *U. dioica* treatment group showed significant reductions in serum glucose, cholesterol, ALT, AST, and triglyceride levels. Furthermore, Moumivand et al. [79] reported that mixtures containing *U. dioica* protected PC12 neuronal cells against glucose-induced toxicity, suggesting additional neuroprotective benefits in diabetic conditions.

Human clinical trials have also demonstrated clinically meaningful outcomes. Kianbakht et al. [80] conducted a double-blind clinical trial (500 mg every 8 h for 3 months) in insulin-dependent patients with type 2 diabetes and reported significant reductions in fasting blood glucose, postprandial glucose, and HbA1c levels (*p* < 0.01) with excellent tolerability. Khalili et al. [81] observed synergistic glycemic and triglyceride-lowering effects in a combined herbal formulation containing *Silybum marianum* and *Boswellia serrata* administered over 90 days, supporting the inclusion of *U. dioica* in polyherbal therapies. A broader Persian clinical study by Mehrzadi et al. [82] further confirmed that an herbal formulation containing *U. dioica*, *Trigonella foenum-graecum*, and *Citrullus colocynthis* significantly reduced fasting blood glucose levels in patients with type 2 diabetes without adverse effects. Additionally, Trasca et al. [83] reviewed Romanian herbal medicine traditions and highlighted *U. dioica* as one of the most prominent medicinal plants with both strong preclinical evidence and emerging clinical support for hypoglycemic activity. Table 7 summarizes key antidiabetic findings for *U. dioica* derived from cellular, animal, and human studies.

**Figure 7:**
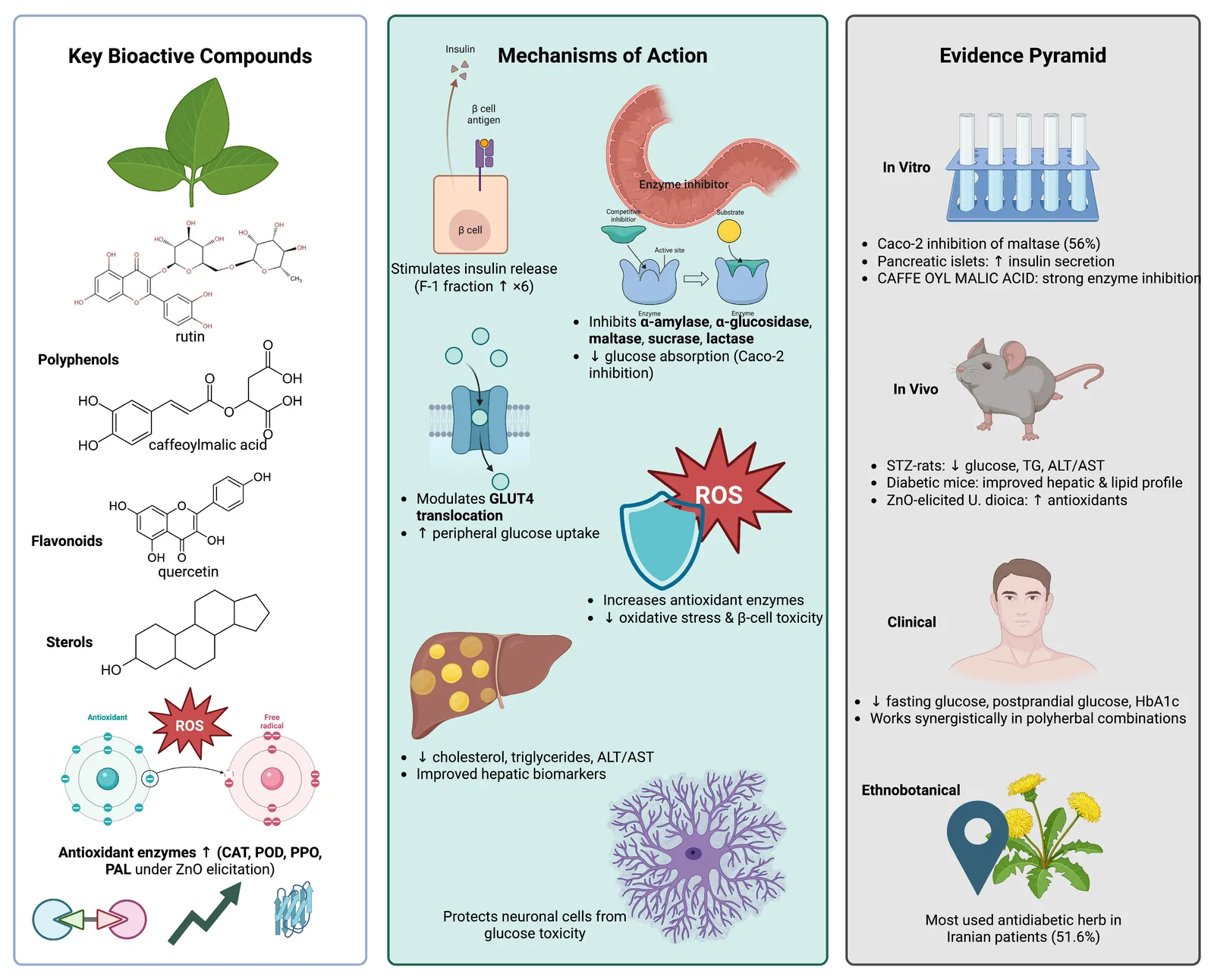
Proposed antidiabetic mechanisms and supporting evidence of *Urtica dioica*, highlighting key bioactive compounds, major mechanisms of action, and evidence from in vitro, in vivo, clinical, and ethnobotanical studies. Awlqadr, F. (14 July 2026), https://BioRender.com/10j3u68.

The antidiabetic activity of *U. dioica* appears to be mediated by multiple complementary mechanisms linked to its diverse phytochemical composition, including polyphenols, flavonoids, phenolic acids, antioxidant metabolites, and phytosterols. Compounds such as rutin, quercetin, and caffeoylmalic acid may contribute to improved glycemic control by enhancing insulin secretion and supporting pancreatic β-cell function. In parallel, *U. dioica* has been reported to inhibit key carbohydrate-digesting enzymes, including α-amylase, α-glucosidase, sucrase, lactase, and maltase, thereby slowing carbohydrate digestion and reducing intestinal glucose absorption. Additional mechanisms include stimulation of GLUT4 translocation and increased peripheral glucose uptake in insulin-sensitive tissues. Its antioxidant properties may further protect β-cells against ROS-mediated damage by scavenging reactive oxygen species and enhancing endogenous antioxidant enzyme activity. Hepatoprotective and lipid-modulating effects have also been described, including reductions in triglycerides, cholesterol, and liver injury markers such as ALT and AST, while neuroprotective effects may help limit glucose-induced neuronal injury. Collectively, evidence from cell-based studies, diabetic animal models, and limited clinical investigations suggests beneficial effects on fasting and postprandial glucose, HbA1c, lipid profile, oxidative stress, and inflammatory status; however, much of the mechanistic evidence remains predominantly preclinical. These integrated mechanisms and evidence levels are summarized in Figure 7.

### 7.5. Antihyperlipidemic Effects

The lipid-lowering potential of *U. dioica* has been extensively documented in both preclinical and clinical studies. Evidence indicates that its bioactive flavonoids (quercetin, kaempferol, and rutin), phenolic acids (caffeic, chlorogenic, and ferulic acids), sterols, and terpenes exert hypolipidemic effects through modulation of lipid metabolism, inhibition of HMG-CoA reductase, improvement of hepatic lipid transport, and reduction in oxidative stress and inflammation. Daher et al. [84] provided early evidence that aqueous *U. dioica* extract (150 mg/kg/day for 30 days) significantly decreased total cholesterol, LDL-C, and LDL/HDL ratios in rats without hepatotoxicity, confirming a direct hypolipidemic effect. Ahangarpour et al. [85] reported improved lipid profiles in fructose-induced insulin-resistant rats treated with 100–200 mg/kg hydroalcoholic extract, suggesting modulation of leptin signaling and VLDL metabolism.

In diabetic models, Eldamaty [86] observed that supplementation with 5–10% nettle powder in the diet significantly reduced triglycerides, total cholesterol, and LDL levels while improving hepatic function, reinforcing its potential as a functional dietary ingredient. At the molecular level, Samakar et al. [64] reviewed evidence indicating that nettle’s antihyperlipidemic activity is mediated through inhibition of HMG-CoA reductase, suppression of lipid peroxidation, and quercetin-mediated reduction in cholesterol biosynthesis. Namazi et al. [87] further demonstrated that treatment with 100 mg/kg ethanolic extract protected against atherosclerosis and hypercholesterolemia in high-fat-fed rats by reducing LDL/HDL ratios and preventing arterial lesion formation. Complementarily, Othman and Nanakali [88] reported that *U. dioica*-derived silver nanoparticles improved serum lipid profiles and antioxidant enzyme activities (SOD and GSH) in hyperlipidemic rats.

Recent studies have expanded these findings. Pedraza et al. [89] reported that inclusion of 1% *U. dioica* in broiler diets decreased total cholesterol, LDL, and triglycerides while enhancing gut microbiota diversity. Similarly, Othmani et al. [90] demonstrated that essential oil of *U. dioica* ameliorated lipid dysregulation and oxidative injury in rats with myocardial infarction through antioxidant and ACE-inhibitory pathways.

Human studies also support these lipid-modulating effects. Tabrizi et al. [91] performed a meta-analysis of 13 clinical trials and confirmed reductions in triglyceride levels and systolic blood pressure following *U. dioica* supplementation, although changes in total cholesterol and LDL cholesterol were modest. Dadvar et al. [92] further demonstrated that combining aerobic training with nettle supplementation improved HDL-C levels and reduced fasting blood glucose in women with diabetes.

Recent mechanistic studies have provided additional insights into gene-level regulation of lipid metabolism. Eren et al. [93] demonstrated that *U. dioica* downregulated key genes involved in triglyceride synthesis and lipid deposition, including LPL, DGAT1, MCP1, and FAS, highlighting its molecular anti-obesity potential. Mohammadzadeh et al. [89] reported that a multi-herbal formulation containing *U. dioica* significantly lowered body mass index (BMI), cholesterol, LDL, and triglyceride levels in obese rats while restoring hepatic and renal biomarkers.

As summarized in Table 8, both preclinical and clinical studies consistently confirm the lipid-lowering and cardioprotective effects of *U. dioica*.

### 7.6. Cardiovascular Benefits

The cardioprotective potential of *U. dioica* is increasingly recognized due to its multifaceted effects on blood pressure regulation, lipid homeostasis, antioxidant defense, and endothelial function. Contemporary evidence from in vitro, in vivo, and clinical studies consistently supports its capacity to attenuate cardiovascular disease (CVD) risk factors and improve cardiac health through multiple molecular and biochemical mechanisms.

Essential oil and ethanolic extracts of *U. dioica* demonstrate powerful antioxidant and vasodilatory properties. Othmani et al. [90] reported that pretreatment with *U. dioica* essential oil (200–300 mg/kg) significantly ameliorated isoproterenol-induced myocardial infarction (MI) in Wistar rats, restoring electrocardiographic abnormalities, reducing lipid peroxidation, and normalizing cardiac enzyme levels (CK-MB, LDH, and troponin I). Molecular docking studies further revealed strong interactions with angiotensin-converting enzyme (ACE) and inflammatory proteins, supporting its antihypertensive and antifibrotic mechanisms. Similarly, Sakla et al. [98] demonstrated that nettle extract modulated oxidative biomarkers such as glutathione S-transferase (GST) and malondialdehyde (MDA) in honeybees, reducing oxidative stress and suggesting potential vascular-protective implications.

Animal studies further corroborate these findings. Namazi et al. [99] confirmed improved left ventricular function and reduced cardiac fibrosis in hyperlipidemic rat models treated with hydroalcoholic extracts of *U. dioica*. Moreover, the combination of nettle extract with physical training exhibited synergistic improvements in endothelial nitric oxide synthase (eNOS) expression and reduced vascular inflammation [92].

Clinical trials also substantiate the cardiovascular benefits of *U. dioica*. Motavasselian et al. [100] conducted a triple-blinded randomized controlled trial involving 36 hypertensive patients receiving *U. dioica* and *Ziziphus jujuba*-based Gaznab syrup (5 mL twice daily for eight weeks). The results indicated significant reductions in systolic (−12 mmHg) and diastolic (−7 mmHg) blood pressure compared with placebo, with no adverse hepatic or renal effects. In another study, Tabrizi et al. [91] performed a meta-analysis of 13 clinical trials showing that *U. dioica* supplementation reduced triglycerides, total cholesterol, and systolic blood pressure while modestly improving HDL-C levels and diastolic function, highlighting its potential in the management of hypertension and dyslipidemia.

The cardioprotective effects of *U. dioica* are mediated through multiple cellular pathways. Phenolic acids (ferulic and caffeic acids) and flavonoids (quercetin and kaempferol) exert antioxidant effects by scavenging free radicals and upregulating antioxidant enzymes such as superoxide dismutase (SOD), catalase, and glutathione peroxidase (GPx). Additionally, *U. dioica* suppresses nuclear factor-κB (NF-κB) and inducible nitric oxide synthase (iNOS), thereby reducing vascular inflammation. ACE inhibition and calcium channel antagonism contribute to reduced vasoconstriction and myocardial workload, while phytosterols enhance lipid clearance by increasing LDL receptor activity. These combined mechanisms alleviate oxidative stress, lower plasma lipid levels, and improve endothelial integrity, thereby protecting against atherosclerosis and ischemic injury.

Chira and Lorenzetti [101] further highlighted that flavonoids present in *U. dioica* reduce neuroinflammation and oxidative damage, indirectly supporting cardiovascular resilience through improved neurovascular coupling. Moreover, Rani et al. [102] observed a significant enhancement of hepatic antioxidant defenses and suppression of oxidative lipid peroxidation in methotrexate-induced hepatotoxic rats, confirming the plant’s systemic antioxidative and vascular-protective effects.

**Figure 8:**
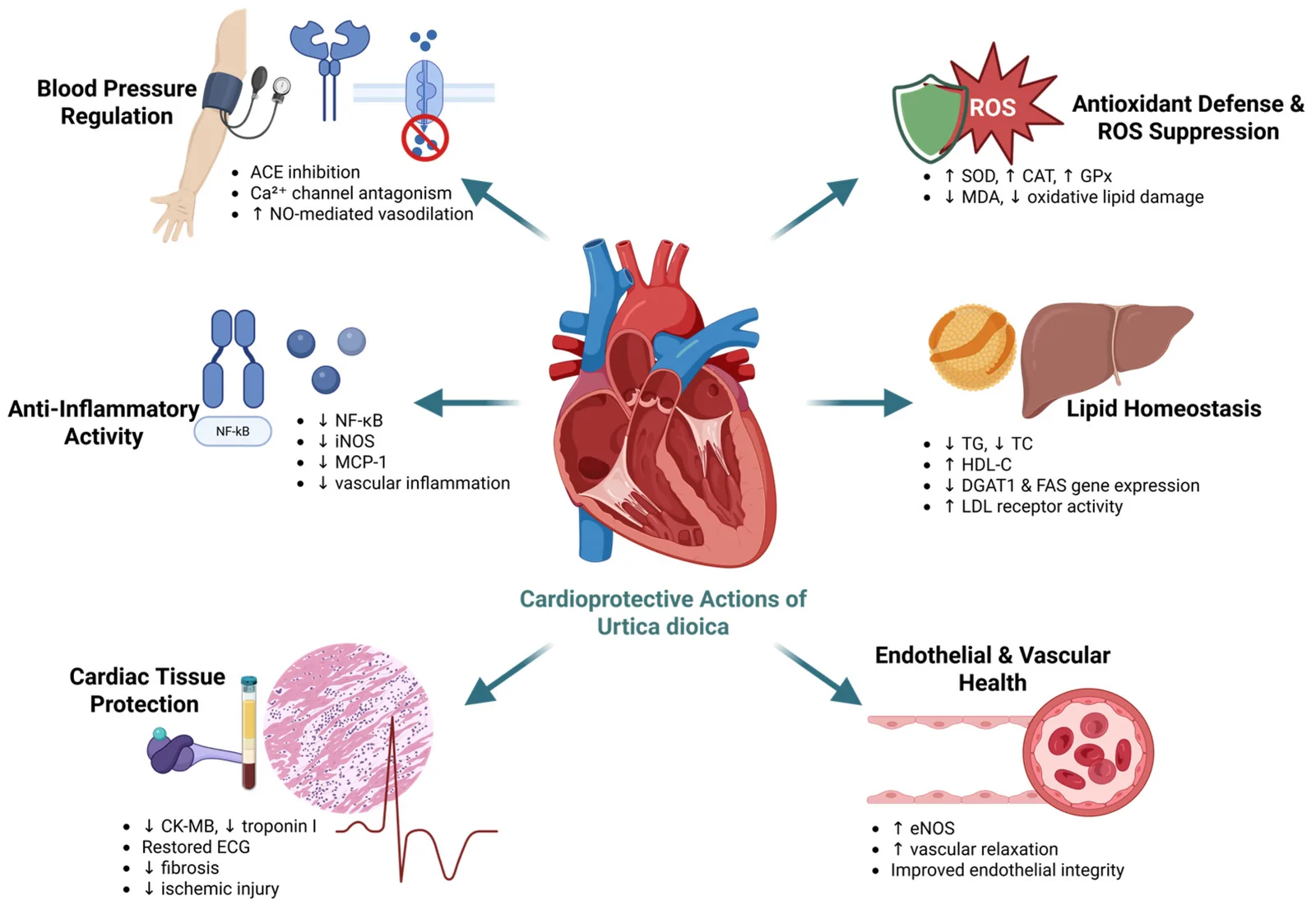
Proposed cardioprotective actions of *Urtica dioica*, including antihypertensive, antioxidant, anti-inflammatory, hypolipidemic, cardiomyocyte-protective, and vasculoprotective effects. Created in BioRender. Awlqadr, F. (14 July 2026), https://BioRender.com/ebbzsic.

The cardioprotective effects of *U. dioica* appear to involve several interconnected mechanisms. Experimental evidence suggests that *U. dioica* may help regulate blood pressure through inhibition of angiotensin-converting enzyme (ACE), modulation of calcium channel activity, and enhancement of nitric oxide (NO)-mediated vasodilation. Its antioxidant activity may further protect cardiovascular tissues by increasing endogenous antioxidant defenses, including GPx, SOD, and CAT, while reducing ROS generation and lipid peroxidation markers such as malondialdehyde (MDA). In addition, anti-inflammatory effects have been associated with suppression of NF-κB signaling and downregulation of inflammatory mediators such as iNOS and MCP-1. Beneficial effects on lipid metabolism have also been reported, including reductions in total cholesterol and triglycerides, increases in HDL cholesterol, and modulation of hepatic lipid-regulating pathways, including FAS, DGAT1, and LDL receptor activity. Cardiac protection has been supported by reductions in biomarkers of myocardial injury, such as troponin I and CK-MB, together with improved ECG parameters, reduced ischemic damage, and attenuation of fibrosis. Furthermore, improved endothelial integrity, enhanced eNOS expression, and better vascular relaxation may contribute to its overall vasculoprotective action. These integrated mechanisms are summarized in Figure 8.

### 7.7. Anti-Cancer Potential

The anticancer activity of *U. dioica* has attracted renewed scientific interest as recent studies continue to reveal its cytotoxic, pro-apoptotic, and gene-modulatory properties. Recent research (2022–2025) confirms its efficacy against various cancer types—including breast, colon, liver, prostate, and ovarian cancers—highlighting its potential as an adjunct or alternative therapeutic agent. *U. dioica* exhibits broad anticancer mechanisms mediated by polyphenols (quercetin, kaempferol, and catechin), sterols, and phenolic acids, which modulate oxidative stress, apoptosis, and tumor signaling pathways.

Karakol et al. [103] demonstrated significant cytotoxic and pro-apoptotic effects of methanolic extracts on MCF-7 and MDA-MB-231 breast cancer cells, with increased Bax/Bcl-2 ratios and reduced cell viability, confirming mitochondrial-mediated apoptosis. Alshammari [104] identified catechin and kaempferol from ethanolic extracts as potent inhibitors of the MTHFD2 enzyme, which plays a crucial role in cancer cell metabolism, highlighting *U. dioica* as a potential source of natural folate-cycle inhibitors.

In hepatocellular and colorectal cancer models, Kardan et al. [105] observed dose-dependent inhibition of HepG2 and HCT-116 cell proliferation. Treatment with methanolic extracts of *U. dioica* upregulated Bax expression and downregulated Bcl-2 expression, suggesting apoptosis through mitochondrial pathways. Similarly, Nafeh et al. [7] demonstrated that *U. dioica* leaf infusion synergized with cisplatin in SKOV-3 ovarian cancer cells, enhancing caspase-3 and caspase-8 activation and PARP cleavage while reducing cell migration and invasion in wound-healing assays.

In prostate cancer models, Asadi-Samani et al. [106] confirmed the antiproliferative activity of ethanolic extracts of *U. dioica* on PC-3 and DU-145 cell lines, achieving IC_50_ values below 100 µg/mL. More recent analyses by Chrysargyris et al. [107] showed that organically cultivated nettle grown under deficit irrigation conditions produced higher phenolic content and exhibited stronger cytotoxicity against colorectal cancer cells compared with conventionally cultivated plants, suggesting that cultivation conditions may directly influence anticancer potency.

At the molecular level, Fattahi et al. [108] reported downregulation of ADA and ODC1 genes along with an increased Bax/Bcl-2 ratio, indicating alterations in polyamine and adenosine metabolism in MCF-7 cells. Recent docking and in silico analyses further support these findings, indicating that *U. dioica*-derived compounds interact with key apoptotic and redox-regulating enzymes.

As summarized in Table 9, numerous studies demonstrate the broad anticancer activity of *U. dioica* across multiple tumor types.

### 7.8. Immunomodulatory Activities

The immunomodulatory potential of *U. dioica* has been extensively validated by recent preclinical and clinical studies, demonstrating its ability to regulate both innate and adaptive immune responses through antioxidant defense, cytokine balance, and modulation of cellular immunity. Its bioactive compounds, particularly chlorogenic acid, quercetin, kaempferol, and lectins, play key roles in balancing inflammatory cascades and enhancing host resistance to infections and physiological stress.

Wójcik-Borowska et al. [25] demonstrated that polyphenolic fractions from *U. dioica* leaves and flowers significantly decreased pro-inflammatory cytokines (IL-1β and IL-6) while upregulating the anti-inflammatory cytokine IL-10 in lipopolysaccharide (LPS)-stimulated fibroblasts, indicating both antioxidant and immune-balancing potential. Abdel-Latif et al. [113] reported that dietary supplementation with *U. dioica* enhanced hematological immune parameters and resistance to *Yersinia ruckeri* infection in rainbow trout, highlighting its potential as a natural immunostimulant in aquaculture.

In another study, Mülleneisen et al. [114] confirmed the safety and efficacy of *U. dioica* in allergen immunotherapy for individuals sensitive to stinging nettle pollen, demonstrating reductions in nasal inflammation and improved clinical tolerance. Rubiyanti et al. [65] also reviewed the dual anti-inflammatory and wound-healing properties of *U. dioica*, reporting inhibition of nitric oxide and TNF-α production, suggesting indirect immunomodulation through the regulation of inflammatory pathways.

Similarly, De Vico et al. [115] demonstrated that dietary supplementation with *U. dioica* enhanced macrophage activity, lysozyme response, and antibody titers in farmed fish, thereby increasing resistance to bacterial infections. Milosevic et al. [116] reported comparable immune enhancement in poultry fed *U. dioica* extracts, including improved leukocyte counts, increased antioxidant capacity, and enhanced immune-related growth performance.

In mammalian models, Beatriz Herrera [117] demonstrated that *U. dioica* extract reversed immunosuppression in malnourished rats by increasing CD4^+^ and CD8^+^ lymphocyte populations and stimulating bone marrow activity. Moreover, Klingelhoefer et al. [118] observed that IDS-23 nettle leaf extract suppressed Th1 cytokines (IL-2 and IFN-γ) while upregulating the Th2 cytokine IL-4 in peripheral blood mononuclear cells (PBMCs), suggesting T-cell rebalancing that may be important in autoimmune regulation.

Recent mechanistic studies further indicate that nettle lectins, particularly *U. dioica* agglutinin (UDA), act as plant-derived mitogens that stimulate IL-2-mediated lymphocyte proliferation and immune signaling. Grauso et al. [119] also reported that polysaccharides and flavonoids present in *U. dioica* exhibit synergistic immunostimulatory and anti-inflammatory effects, supporting its adaptogenic properties.

**Figure 9:**
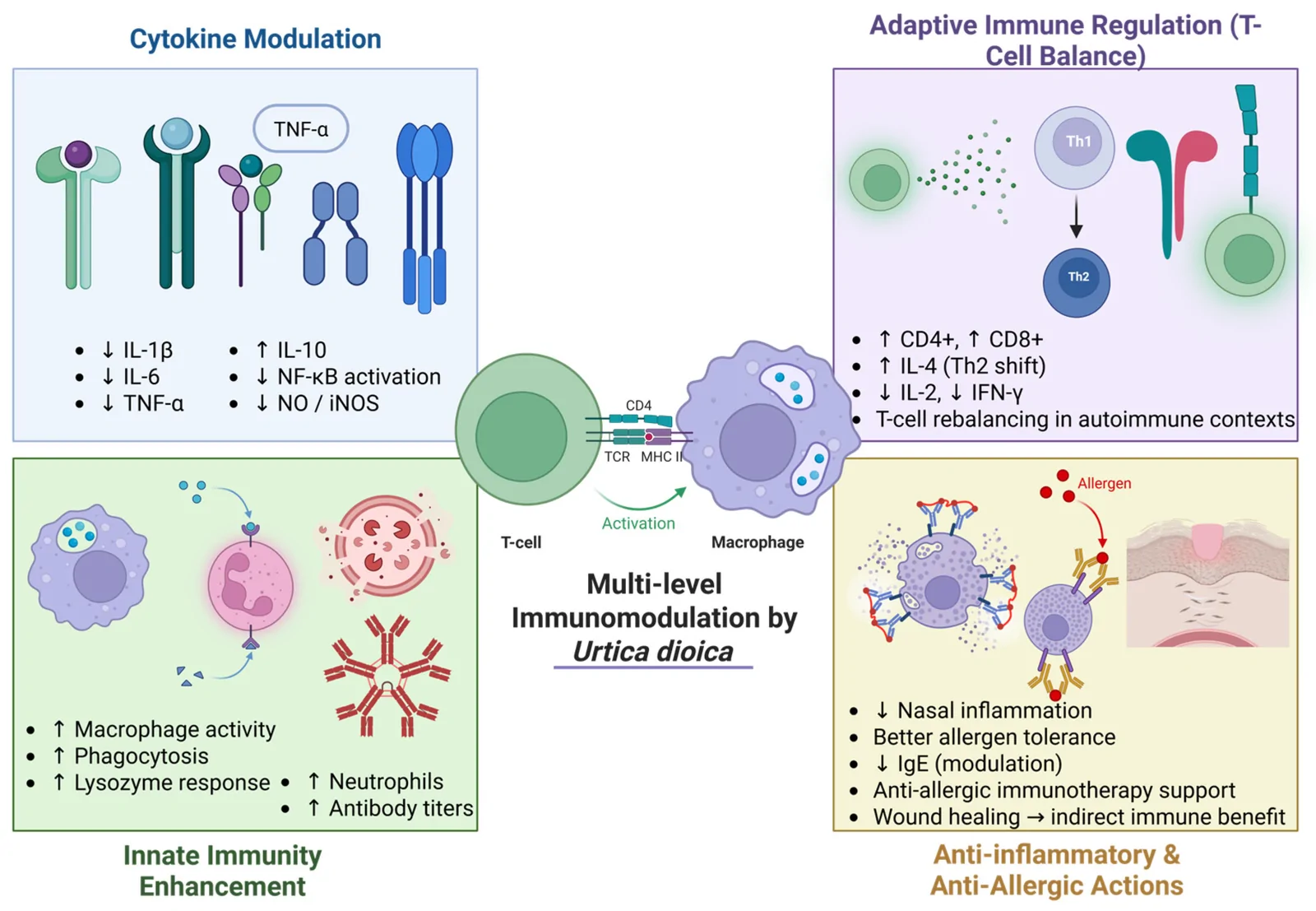
Proposed immunomodulatory effects of *Urtica dioica*, including cytokine modulation, enhancement of innate immune responses, regulation of adaptive immunity, and anti-inflammatory/anti-allergic actions. Arrows indicate directional activation, modulation, or regulatory relationships among immune components, whereas the upward and downward arrows denote increased and decreased responses, respectively. The color-coded panels distinguish the major immunomodulatory pathways: blue represents cytokine modulation, purple represents adaptive immune regulation, green represents innate immunity enhancement, and yellow represents anti-inflammatory and anti-allergic actions. Created in BioRender. Awlqadr, F. (23 July 2026), https://BioRender.com/vslbk7v.

*U. dioica* appears to exert immunomodulatory effects through coordinated actions on both innate and adaptive immunity. Reported mechanisms include cytokine modulation, characterized by reductions in pro-inflammatory mediators such as IL-1β, IL-6, and TNF-α, together with increased IL-10 production and suppression of NF-κB-related inflammatory signaling. In innate immunity, *U. dioica* has been associated with enhanced macrophage activity, phagocytosis, lysozyme response, neutrophil function, and antibody production, suggesting improved first-line immune defense. At the adaptive level, it may contribute to rebalancing T-cell responses, including modulation of Th1/Th2 pathways, increased CD4^+^ and CD8^+^ T-cell activity, elevated IL-4, and reduced IL-2 and IFN-γ in certain inflammatory or autoimmune contexts. These combined effects may underlie its anti-inflammatory, anti-allergic, and immune-stabilizing properties, including reduced nasal inflammation, improved allergen tolerance, and supportive effects on wound healing. These effects are summarized in Figure 9.

As outlined in Table 10, *U. dioica* exhibits strong immunomodulatory activity across diverse experimental models.

## 8. Gene Expression and Molecular Mechanisms

*U. dioica* exerts profound regulatory effects on cellular gene expression, particularly on genes involved in apoptosis, inflammation, oxidative stress, and signal transduction. Nafeh et al. [7] reported that nettle leaf infusion significantly enhanced cisplatin-induced apoptosis in ovarian cancer SKOV-3 cells by upregulating CASP3 and CASP8, promoting PARP cleavage and DNA fragmentation, thereby activating the extrinsic caspase-dependent pathway. Similarly, Rani et al. [102] demonstrated hepatoprotective effects in methotrexate-induced rats by increasing the activities of SOD and GPx while reducing serum transaminase levels and histopathological damage, suggesting antioxidative and anti-inflammatory regulation through NF-κB inhibition. Additionally, Sardari et al. [121] reported that ZnO-nanoparticle elicitation in *U. dioica* callus significantly enhanced the activities of PAL, POD, CAT, and PPO enzymes, along with increased phenolic accumulation (quercetin and total phenolics). These findings indicate that nanoparticle treatments can stimulate oxidative-stress-responsive genes that strengthen plant antioxidant metabolism, suggesting potential enhancement of antioxidant gene pathways for therapeutic applications.

Modulation of the PI3K/AKT/eNOS and MAPK signaling pathways represents another important mechanism of action. AL-Attabi et al. [122] reported that nettle extract suppressed the PI3K/AKT/eNOS signaling axis and vascular endothelial growth factor (VEGF) expression in DU-145 prostate cancer cells, leading to anti-angiogenic and pro-apoptotic effects. Similar outcomes were observed in diabetic models, where *U. dioica* improved hippocampal insulin signaling and downregulated oxidative and inflammatory markers such as IL-1β and TNF-α [123]. These findings illustrate the plant’s capacity to restore metabolic signaling homeostasis through the AKT and MAPK pathways.

Epigenetic regulation is emerging as an additional mechanism underlying the biological activity of *U. dioica*. Polyphenolic constituents such as quercetin and chlorogenic acid [124] can interact with chromatin-modifying enzymes and histone acetylation regulators, thereby modulating genes associated with lipid and glucose metabolism. This suggests potential roles as epigenetic modulators in metabolic disorders such as diabetic hepatopathy. In cancer research, such epigenetic modulation may also contribute to enhanced apoptosis sensitivity and improved regulation of oxidative stress.

**Figure 10:**
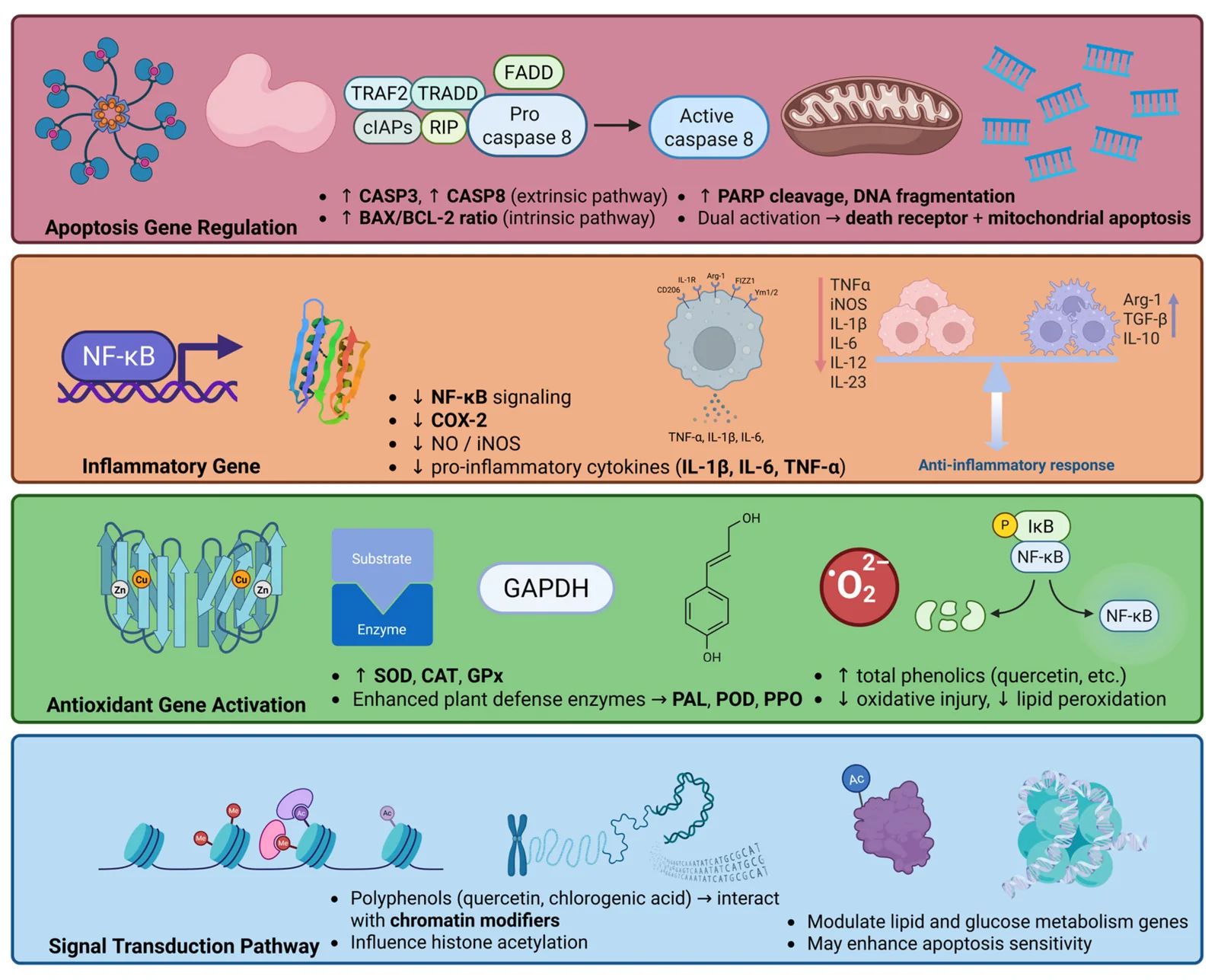
Proposed gene expression and signaling mechanisms modulated by *Urtica dioica*, including apoptosis regulation, inflammatory gene suppression, antioxidant gene activation, and signal transduction and epigenetic effects. Arrows indicate the direction of molecular signaling, pathway activation, or progression toward downstream cellular effects, whereas the upward and downward arrows represent increased and decreased expression or activity, respectively. The color-coded panels distinguish the principal regulatory mechanisms: pink represents apoptosis-related gene regulation, orange represents inflammatory gene regulation, green represents antioxidant gene activation, and blue represents chromatin-associated signaling and metabolic gene modulation. Created in BioRender. Awlqadr, F. (14 July 2026), https://BioRender.com/toaz2lu.

Collectively, these studies demonstrate that *U. dioica* orchestrates a multi-target molecular network involving activation of apoptosis-related genes (BAX, CASP3, CASP8), suppression of inflammatory mediators (NF-κB, COX-2, IL-6), enhancement of antioxidant defense genes (SOD, CAT, GPx), and regulation of metabolic and angiogenic signaling pathways (PI3K/AKT/eNOS and MAPK). However, important knowledge gaps remain regarding dose-dependent gene expression kinetics, tissue-specific responses, and comprehensive epigenomic mapping. Future omics-based and clinical investigations will be essential to fully define the molecular signatures of *U. dioica* in both cancer and metabolic disorders. *U. dioica* has been reported to modulate gene expression through multiple interconnected molecular pathways. Experimental evidence suggests that its extracts may promote apoptosis through both extrinsic and intrinsic mechanisms, including activation of caspase-8 and caspase-3 and an increase in the BAX/BCL-2 ratio, culminating in mitochondrial dysfunction, PARP cleavage, and DNA fragmentation. In parallel, *U. dioica* appears to suppress inflammatory gene expression by inhibiting NF-κB signaling and downregulating downstream mediators such as COX-2, iNOS/NO, and pro-inflammatory cytokines, including IL-1β, IL-6, TNF-α, IL-12, and IL-23, while increasing anti-inflammatory markers in some models, such as Arg-1, TGF-β, and IL-10. It also enhances antioxidant defense by increasing the activity or expression of antioxidant enzymes, including SOD, CAT, and GPx, and by supporting phenolic-mediated protective responses that reduce oxidative injury and lipid peroxidation. In addition, polyphenolic constituents such as quercetin and chlorogenic acid may interact with chromatin-associated regulatory mechanisms and influence genes involved in lipid metabolism, glucose homeostasis, and apoptosis sensitivity. These integrated molecular effects are summarized in Figure 10.

## 9. Future Perspectives

Future research on *U. dioica* should focus on transforming it from a traditional herbal remedy into a scientifically standardized and widely recognized therapeutic resource. Although its antioxidant, anti-inflammatory, antidiabetic, hepatoprotective, and anticancer properties are well documented, further attention is required regarding extract standardization, innovative delivery systems, and deeper molecular characterization.

The development of standardized extracts is particularly important because the bioactive composition of the plant, especially quercetin, chlorogenic acid, caffeoylmalic acid, and *U. dioica* agglutinin, varies depending on environmental conditions and extraction methods. Establishing pharmacopeial quality specifications and applying green extraction technologies, including pressurized liquid extraction and supercritical CO_2_ extraction, could ensure reproducibility and the production of high-quality formulations.

Integration of *U. dioica* into functional foods and nutraceutical products also represents a promising strategy for preventive healthcare. The leaves and seeds contain high levels of vitamins, minerals, and phenolic compounds that support metabolic control, lipid regulation, and immune function. Fortification of teas, dairy products, and protein-based beverages with nettle extracts may improve consumer acceptance, particularly when combined with nanoencapsulation technologies that protect bioactive compounds during processing and digestion.

The pharmaceutical potential of *U. dioica* remains substantial. Bioactive fractions have been shown to modulate signaling pathways such as NF-κB, MAPK, and PI3K/AKT, suppress pro-inflammatory mediators, and induce apoptosis in cancer cells. *U. dioica* agglutinin (UDA) demonstrates immunomodulatory and antiviral activity, while phenolic-rich extracts exhibit antioxidant and anti-angiogenic properties, indicating broad therapeutic potential. However, dose optimization, pharmacokinetic characterization, and safety assessments are necessary before clinical application.

Advances in omics technologies, including transcriptomics, proteomics, and metabolomics, will be crucial for identifying the specific genes, proteins, and metabolites responsible for these biological effects. Integrating such datasets may reveal regulatory networks linking antioxidant defense, apoptosis, and metabolic regulation, thereby supporting the development of personalized nutraceuticals or phytopharmaceuticals. Additionally, nanotechnology may enhance delivery efficiency; encapsulation of nettle phytochemicals into liposomes, nanogels, or lipid nanoparticles has already improved stability, solubility, and controlled release, while nanoparticle elicitation may further increase secondary metabolite production in plant systems.

Global regulatory harmonization will also be essential for broader acceptance of *U. dioica*-based products. Establishing international quality standards, comprehensive safety data, and evidence-based health claims will enable these products to enter pharmaceutical and functional food markets with greater confidence. Collaboration among researchers, regulatory agencies, and industry stakeholders may accelerate approval processes and ensure compliance with FDA, EMA, and EFSA regulatory frameworks.

## 10. Conclusions

*U. dioica* represents a remarkable medicinal plant whose therapeutic potential spans centuries of traditional use and modern pharmacological validation. Extensive research has demonstrated that the efficacy of its bioactive compounds, including flavonoids, phenolic acids, lignans, sterols, and fatty acids, depends largely on optimized extraction techniques and precise chemical characterization. Recent technological advances, such as UAE, MAE, PLE, and SFE, have significantly improved extraction yield, purity, and bioactivity compared with conventional maceration and Soxhlet methods. These standardized and environmentally sustainable approaches enable reproducible identification of key constituents such as quercetin, chlorogenic acid, β-sitosterol, and *U. dioica* agglutinin, which underpin the plant’s multifunctional biological activities.

Across experimental and clinical models, *U. dioica* demonstrates a broad spectrum of health benefits. Its potent antioxidant properties neutralize reactive oxygen species, while anti-inflammatory effects occur through inhibition of NF-κB and COX-2 pathways. Additionally, antimicrobial and antiviral activities are mediated by lectins and polyphenols, and metabolic regulation improves glucose and lipid homeostasis. These synergistic mechanisms contribute to protective effects against diabetes, hyperlipidemia, cardiovascular diseases, and oxidative stress-related degenerative disorders.

Integration of nettle-derived bioactive compounds into nutraceuticals, herbal formulations, and functional foods has demonstrated promising benefits in promoting immune balance, organ protection, and systemic redox stability. At the molecular level, *U. dioica* has emerged as a promising regulator of gene expression and cellular signaling pathways. Recent transcriptomic and proteomic studies indicate that phenolic-rich extracts regulate apoptosis-related genes (↑BAX, ↑caspase-3, ↓BCL-2), suppress pro-inflammatory cytokine transcription, and upregulate antioxidant defense genes (SOD, CAT, and GPx). Furthermore, modulation of key signaling pathways, including NF-κB, PI3K/AKT, and MAPK, links its bioactivity to improved mitochondrial function and reduced inflammatory stress, establishing a strong mechanistic basis for its therapeutic potential. Emerging evidence also suggests possible epigenetic regulation through histone modification and DNA methylation, further expanding its relevance in cancer prevention and metabolic disease management.

## Figures and Tables

**Table 1:** Mineral and trace metal concentrations in *Urtica dioica* [20,21,22].

Element	Element	Symbol	Concentration (mg/kg dw)
Bulk Elements (Macro-minerals)	Calcium	Ca	16,500–24,890
	Potassium	K	25,340–29,210
	Magnesium	Mg	4400–9020
	Phosphorus	P	5100–6600
	Sodium	Na	170.8–236.4
	Iron	Fe	267–442
	Strontium	Sr	156.78
	Manganese	Mn	38.8–67.6
	Barium	Ba	31.16
	Zinc	Zn	19.5–31.1
Trace Elements (Micro-minerals)	Copper	Cu	8.7–16.2
	Nickel	Ni	0.08–12.2
	Cerium	Ce	1.57
	Chromium	Cr	0.34–1.0
	Lanthanum	La	0.70
	Cobalt	Co	0.50
	Nickel	Ni	2.0
Polluting/Potentially Toxic Elements	Chromium	Cr	1.77
	Barium	Ba	37.5

**Table 2:** Comprehensive chemical composition of *Urtica dioica* leaves identified by UHPLC-HRMS/MS [23,24,25].

Phytochemical Class	Identified Compounds
Hydroxycinnamic acids and derivatives	Caffeic acid; *p*-Coumaric acid; Caffeic acid hexoside; *p*-Coumaric acid hexoside; 2-Caffeoylisocitric acid; 2-p-Coumaroylisocitric acid; 2-Feruloylisocitric acid; 2-Caffeoylisocitric acid cyclodimer
Simple phenolics and coumarins	Dihydroxybenzoic acid hexosides (isomers); Hydroxytyrosol hexoside; Esculin
C-glycosylated flavonoids	Luteolin-6-C-hexoside; Apigenin-6,8-di-C-hexoside; C-glycosyl-O-deoxyhexosyl luteolin; C-glycosyl-O-deoxyhexosyl apigenin; C-glycosyl-O-deoxyhexosyl diosmetin
HMG-conjugated flavonoids (Statin-like)	Luteolin-O-deoxyhexosyl-(HMG)-C-hexoside; Apigenin-O-deoxyhexosyl-(HMG)-C-hexoside; Methylluteolin-O-deoxyhexosyl-(HMG)-C-hexoside; Methylluteolin-(HMG)-C-hexoside
Lignans and glycosylated lignans	7-Hydroxylariciresinol-7-O-hexoside; Neo-olivil-9-O-hexoside
Oxylipins (PUFA-derived lipids)	Trihydroxy-octadecadienoic acid; Trihydroxy-octadecenoic acid; Dihydroxy-octadecatrienoic acid; Hydroxy-oxo-octadecatrienoic acid; Dioxo-octadecatrienoic acid; Hydroxy-octadecatrienoic acid; Hydroxy-octadecenoic acid
Minor/Newly proposed metabolites	Caffeoyl-methylisocitric acid; Several unassigned but structurally elucidated ions

Abbreviations used: HMG, 3-hydroxy-3-methylglutaryl; PUFA, polyunsaturated fatty acid.

**Table 3:** Comparative advantages of modern vs. conventional extraction methods.

Extraction Method	Solvent Type and Safety	Typical Temperature/Time	Plant Part Used	Major Bioactive Compounds Extracted	Yield and Efficiency	Cost and Scalability	Limitations	References
Maceration (conventional)	Ethanol, methanol, water (toxic solvents possible)	25–60 °C/6–72 h	Leaves, roots	Chlorogenic acid, rutin, caffeic acid, flavonoids	Moderate (50–80 mg GAE/g)	Low cost, simple equipment	Long time, low diffusion, solvent waste	[26,27]
Soxhlet extraction (conventional)	Ethanol, hexane, methanol	60–80 °C/4–8 h	Whole plant	Fatty acids, sterols, triterpenes	High for thermostable compound	Scalable but energy-intensive	Thermal degradation, solvent residue	[28]
Hydrodistillation (conventional)	Water (safe)	95–100 °C/2–4 h	Leaves, Stems	β-caryophyllene, α-terpineol, carvacrol	Low yield (0.2–0.5%)	Simple apparatus	Hydrolysis of terpenes	[7]
UAE	Water, ethanol, NADES (food-grade)	30–50 °C/20–30 min	Leaves	Quercetin, chlorogenic acid, luteolin, caffeic acid	High (132 mg GAE/g)	Low energy and solvent use	Equipment cost	[27,29]
MAE	water, ethanol, NADES	60–90 °C/10–17 min	Leaves	Quercetin, rutin, kaempferol	High (TPC ≈ 108 mg GAE/g)	Fast and efficient	Possible overheating	[15]
SFE	CO_2_ + Ethanol (GRAS)	40–60 °C/60–120 min	Leaves, seeds	β-sitosterol, terpenes, carotenoids	Moderate (0.2–0.5%)	Solvent-free, selective	High equipment cost	[28]
PLE	Ethanol, Water (safe)	100–200 °C/5–20 min	Roots, leaves	Phenolic acids, triterpenes, Isorhamnetin	Very high (1.6% yield; 50 mg/100 g β-sitosterol)	Industrially adaptable	Needs pressure control	[29]
DES-UAE	Food-grade NADES (citric acid + maltose)	70 °C/30 min	Whole plant	Quercetin-3-O-rutinoside, caffeoylquinic acid	Very high (TPC 2423 mg GAE/100 g)	Green, non-toxic, recyclable	Viscosity limits diffusion	[30]

Abbreviations used: DES, deep eutectic solvent; GAE, gallic acid equivalents; GRAS, generally recognized as safe; HMG, 3-hydroxy-3-methylglutaryl; MAE, microwave-assisted extraction; NADES, natural deep eutectic solvents; PLE, pressurized liquid extraction; SFE, supercritical fluid extraction; TPC, total phenolic content; UAE, ultrasound-assisted extraction.

**Table 4:** Comparison of chromatographic techniques for *Urtica dioica*.

Method	Analyte Type	Typical Conditions	Advantages	Limitations	References
HPLC–DAD	Phenolic acids, flavonoids	C18 column, 25 °C, 40 min gradient	Quantitative, reproducible	Moderate solvent use	[47,48]
UPLC–MS	Small polar metabolites	Sub-2 µm column, 10 min run	Fast, high resolution	High cost	[52]
GC–MS	Volatiles, fatty acids	60–250 °C program	Identifies aromatics & lipids	Requires derivatization	[49,50]

Abbreviations used: GC-MS, gas chromatography–mass spectrometry; HPLC-DAD, high-performance liquid chromatography with diode-array detection; UPLC-MS, ultra-performance liquid chromatography-mass spectrometry.

**Table 5:** Comparison of spectroscopic techniques for *Urtica dioica*.

Technique	Purpose	Typical Parameters	Advantages	Limitations	References
FTIR	Functional group identification	4000–400 cm^−1^ range	Rapid, non-destructive	Low quantitative power	[47,49]
^1^H/^13^C NMR	Structural confirmation	400–600 MHz, CD_3_OD solvent	Definitive structure	High sample need	[47,52]
LC–MS/MS	Molecular mass and quantitation	ESI ±, *m*/*z* 50–1000	High sensitivity	Costly instrumentation	[47,52]

Abbreviations used: ^1^H/^13^C NMR, proton and carbon-13 nuclear magnetic resonance spectroscopy; CD_3_OD, deuterated methanol; ESI, electrospray ionization; FTIR, Fourier transform infrared spectroscopy; LC–MS/MS, liquid chromatography–tandem mass spectrometry; MHz, megahertz.

**Table 6:** Antimicrobial and antiviral effects of *Urtica dioica*.

Model/Condition	Part/Preparation	Key Antimicrobial/Antiviral Endpoints (Dose/Conditions)	Reference
In vitro antibacterial & antifungal (food and clinical strains)	Leaf aqueous & ethanolic extracts Review paper	Strong inhibition of *E. coli* and *S. aureus* (all leaf extracts); moderate inhibition of *L. monocytogenes* and *P. aeruginosa*; *Candida glabrata* highly sensitive to aqueous leaf extract	[70]
In vitro antibacterial	Leaf & root phenolic extracts (LC-MS/MS characterized)	Broad-spectrum activity vs. *E. coli*, *S. aureus*, *Klebsiella* spp., *P. aeruginosa*; MIC 0.07–0.15 mg/mL, bactericidal at higher doses	[57]
In vitro antiviral (foodborne virus model)	Cold-brewed aqueous leaf extract	Nearly complete inactivation of norovirus at non-toxic antioxidant concentrations	[38]
Green nanotechnology—antibacterial and antifungal	Leaf-extract-mediated selenium nanoparticles	Strong activity vs. *E. coli*, *P. aeruginosa*, *S. aureus*, *B. subtilis*; MIC 31.25–500 µg/mL; potent antifungal effect vs. *Candida* and *Aspergillus* spp.	[68]
Food preservation model (shrimp, refrigerated storage)	PLA films incorporated with nettle-AgNPs	Suppressed growth of *E. coli*, *S. aureus*, *A. niger*; shelf-life extended to 12 days	[67]
In vitro antibacterial (food-poisoning bacteria)	Seed extracts	Large inhibition zones vs. *B. subtilis* and *S. aureus*; moderate activity vs. *E. coli*; MIC_50_ 14–271 mg/mL	[67]
In vitro antibacterial (broad panel)	Root extract (70% acetone)	Active vs. 29 microbial strains including *Micrococcus*, *Enterococcus*, *Staphylococcus* spp.	[74]
In vitro antibacterial (oral pathogens)	Root acetone extract	Significant inhibition of *Streptococcus* mutans, *E. faecalis*, *K. pneumoniae*; no antifungal activity	[74]

Abbreviations used: AgNPs, silver nanoparticles; LC-MS/MS, liquid chromatography-tandem mass spectrometry; MIC, Minimum inhibitory concentration; PLA, polylactic acid.

**Table 7:** Antidiabetic findings for *U. dioica* from cellular, animal, and human studies.

Study Type	Model/Dose	Key Findings	Reference
In vivo	STZ-rats, 100 mg/kg	↓ glucose, cholesterol, TG, ALT/AST	[78]
In vitro/in vivo	Diabetic mice	Caffeoylmalic acid ↓ blood glucose, improved lipid and hepatic markers	[77]
In vitro	Pancreatic islets	F-1 fraction ↑ insulin × 6; ↓ glucose	[75]
In vivo	OGTT model, 250 mg/kg	↓ Glycemia by 33% via ↓ intestinal absorption	[76]
Clinical	Type 2 DM (n = 46)	↓ Fasting & postprandial glucose, HbA1c	[75]
Clinical	Type 2 DM (n = 60)	↓ Glucose and TG with mixed herbs	[80]
Clinical	Type 2 DM (n = 60)	Herbal combo (incl. *U. dioica*) ↓ fasting glucose	[81]

Abbreviations used: ALT, alanine aminotransferase; AST, aspartate aminotransferase; DM, diabetes mellitus; HbA1c, hemoglobin A1c; OGTT, oral glucose tolerance test; STZ, streptozotocin; TG, triglyceride.

**Table 8:** Studies on antihyperlipidemic effects of *Urtica dioica*.

Study Type	Model/Dose	Key Findings	Reference
In vivo	Rats, 150 mg/kg/day, 30 days	↓ TC, LDL, apoB; improved HDL	[84]
In vivo	Rats, 100–200 mg/kg/day	↓ LDL, leptin, improved lipid ratio	[85]
In vivo	Diabetic rats + 5–10% powder	↓ TG, LDL, improved liver/kidney	[86]
Review	Mechanistic review	HMG-CoA reductase inhibition, antioxidant	[64]
In vivo	HFD rats, 100 mg/kg	↓ LDL/HDL ratio, less atherosclerosis	[87]
In vivo	Rats, AgNPs 15 mg/kg	↓ TC, TG, ↑ SOD, GSH	[88]
Clinical meta-analysis	13 trials, n = 486	↓ TG (−26.9 mg/dL), ↓ SBP	[91]
Clinical	Type II diabetic women	↑ HDL, ↓ FBG	[92]
In vivo	Broiler chickens, 1% diet	↓ TC, LDL, TG, ↑ Lactobacillus	[89]
In vivo	MI rats, 200–300 mg/kg	↓ Lipid peroxidation, improved profile	[90]
In vivo	HFD rats + GUTAC formula	↓ BMI, LDL, TG, improved liver	[94]
In vitro/in silico	Gene expression, docking	↓ LPL, DGAT1, MCP1	[93]
In vivo	Ovariectomized rats, 200 mg/kg	↓ TG, VLDL, improved HDL/LDL	[95]
In vivo	Poultry layers, 1% nettle	↓ Cholesterol, improved FCR	[96]
Review	Systematic herbal tea review	Validated lipid-lowering role	[97]

Abbreviations used: AgNPs, silver nanoparticles; apoB, apolipoprotein B; BMI, body mass index; DGAT1, diacylglycerol O-acyltransferase 1; FBG, fasting blood glucose; FCR, feed conversion ratio; GSH, glutathione; HDL, high-density lipoprotein; HFD, high-fat diet; HMG-CoA, 3-hydroxy-3-methylglutaryl coenzyme A; LDL, low-density lipoprotein; LPL, lipoprotein lipase; MCP1, monocyte chemoattractant protein-1; MI, myocardial infarction; SBP, systolic blood pressure; SOD, superoxide dismutase; TC, total cholesterol; TG, triglyceride; VLDL, very low-density lipoprotein.

**Table 9:** Studies on anticancer effects of *Urtica dioica* (2020–2025).

Cancer Type	Study Type	Model/Dose	Key Findings	Reference
Breast	In vitro	MCF-7, MDA-MB-231/100–400 µg/mL	Apoptosis via Bax/Bcl-2 modulation	[103]
Breast	In vitro and in silico	Ethanolic extract/48 h	MTHFD2 inhibition, reduced viability	[104]
Ovarian	In vitro	SKOV-3 + cisplatin/20–100 µg/mL	Enhanced apoptosis and migration inhibition	[109]
Colon	In vitro	HCT-116/400 µg/mL	Apoptosis via caspase pathway	[105]
Liver	In vitro	HepG2/410 µg/mL	Bax/Bcl-2 modulation, apoptosis induction	[105]
Prostate	In vitro	PC-3, DU145/100 µg/mL	Dose-dependent cytotoxicity	[106]
Bladder	In vitro	T24/50–200 µg/mL	Concentration-dependent apoptosis	[110]
Breast	In vitro	MCF-7/aqueous extract	DNA fragmentation, caspase-3 activation	[111]
Hepatocellular	In vitro	HepG2/200 µg/mL	ROS reduction and anti-angiogenic effect	[112]
Colorectal	In vitro	HT-29/100 µg/mL	Reduced proliferation, apoptosis activation	[107]
Ovarian	In vitro	PAI-1/50 µg/mL	Caspase-dependent apoptosis	[7]
Breast	In vitro	MCF-7/2 mg/mL extract	Mitochondrial pathway activation	[108]
Cervical	In vitro	HeLa/150 µg/mL	Induced ROS-mediated apoptosis	[93]
Liver	In silico	Docking study	Kaempferol binds apoptosis targets	[104]
Colon	In vitro	Caco-2/200 µg/mL	Antioxidant and cytotoxic synergy	[107]
Breast	In vitro	MCF-7/polyphenolic extract	Inhibited PI3K/Akt signaling	[103]

Abbreviations used: AGS, Human gastric adenocarcinoma cell line; Caco-2, colorectal adenocarcinoma cell line 2; CDX1/2, caudal-type homeobox 1 and 2; DNA, deoxyribonucleic acid; DU145, Duke University 145; HeLa, Henrietta Lacks; MCF, Michigan Cancer Foundation; MDA-MB-231, MD Anderson Metastatic Breast Cancer-231; MTHFD2, methylenetetrahydrofolate dehydrogenase 2; HCT-116, human colorectal carcinoma cell line 116; HepG2, human hepatocellular carcinoma cell line 2; MKN-45, human gastric cancer cell line 45; NF-κB, nuclear factor kappa-light-chain-enhancer of activated B cells; PAI-1, plasminogen activator inhibitor-1; PC-3, prostate cancer cell line 3; ROS, reactive oxygen species; SKOV-3, SK ovary 3.

**Table 10:** Key studies on immunomodulatory effects of *Urtica dioica*.

Study Type	Model/Dose	Key Findings	Reference
In vitro	Human fibroblasts, 100 µg/mL	↓ IL-1β, IL-6; ↑ IL-10	[25]
Review	Rainbow trout	↑ Immune resistance, ↓ infection mortality	[113]
Clinical	34 patients (AIT therapy)	↓ Nasal inflammation, improved tolerance	[114]
Review	In vitro/in vivo	↓ TNF-α, NO; wound-healing enhancement	[65]
Review	Fish model	↑ Macrophage, lysozyme, antibody titer	[115]
In vivo	Poultry diet (1%)	↑ Leukocytes, immune performance	[116]
In vivo	Malnourished rats, 0.2 g/mL	↑ CD4^+^, CD8^+^, RBC, WBC	[117]
In vitro	Human PBMCs	↓ IL-2, IFN-γ; ↑ IL-4 (Th2 shift)	[118]
Review	Human overview	Immunostimulant, adaptogen	[119]
In vivo	Wistar rats	↑ Macrophage and neutrophil counts	[117]
In vivo	Broiler chickens	↑ Immunoglobulin, antioxidant enzymes	[116]
In vivo	Fish immune challenge	↑ Lysozyme, phagocytosis	[120]
Review	General review	Reduced oxidative cytokine damage	[119]

Abbreviations used: AIT, allergen immunotherapy; CD4^+^, CD4-positive T cells; CD8^+^, CD8-positive T cells; IFN-γ, interferon-gamma; IL-1β, interleukin-1 beta; IL-2, interleukin-2; IL-4, interleukin-4; IL-6; interleukin-6; IL-10, interleukin-10; NO, nitric oxide; PBMCs, peripheral blood mononuclear cells; RBC, Red Blood cells; TNF-α, tumor necrosis factor alpha; WBC, white blood cells.

## Data Availability

No new data were created or analyzed in this study. Data sharing is not applicable to this article.

## Abbreviations

The following abbreviations are used in this manuscript:

^1^H/^13^C NMR	Proton and carbon-13 nuclear magnetic resonance spectroscopy
AgNPs	Silver nanoparticles
AIT	Allergen immunotherapy
ALT	Alanine aminotransferase
apoB	Apolipoprotein B
AST	Aspartate aminotransferase
BMI	Body mass index
Caco-2	Colorectal adenocarcinoma cell line 2
CD4^+^	CD4-positive T cells
CD8^+^	CD8-positive T cells
CD_3_OD	Deuterated methanol
CDX1/2	Caudal-type homeobox 1 and 2
DES	Deep eutectic solvent
DGAT1	Diacylglycerol O-acyltransferase 1
DNA	Deoxyribonucleic acid
DU145	Duke University 145
ESI	Electrospray ionization
FBG	Fasting blood glucose
FCR	Feed conversion ratio
FTIR	Fourier transform infrared spectroscopy
GAE	Gallic acid equivalents
GC-MS	Gas chromatography-mass spectrometry
GRAS	Generally recognized as safe
GSH	Glutathione
HCT-116	Human colorectal carcinoma cell line 116
HFD	High fat diet
HMG	3-hydroxy-3-methylglutaryl
HMG-CoA	3-hydroxy-3-methylglutaryl coenzyme A
HepG2	Human hepatocellular carcinoma cell line 2
HeLa	Henrietta Lacks
HDL	High-density lipoprotein
HPLC-DAD	High-performance liquid chromatography with diode-array detection
IFN	Interferon
IFN-γ	Interferon-gamma
IL-1β	Interleukin-1 beta
IL-2	Interleukin-2
IL-4	Interleukin-4
IL-6	Interleukin-6
IL-10	Interleukin-10
LC–MS/MS	Liquid chromatography–tandem mass spectrometry
LDL	Low-density lipoprotein
LPL	Lipoprotein lipase
MAE	Microwave assisted extraction
MCF	Michigan Cancer Foundation
MCP1	Monocyte chemoattractant protein-1
MDA-MB-231	MD Anderson metastatic breast cancer-231
MHz	Megahertz
MIC	Minimum inhibitory concentration
MI	Myocardial infarction
MKN-45	Human gastric cancer cell line 45
MTHFD2	Methylenetetrahydrofolate dehydrogenase 2
NADES	Natural deep eutectic solvents
NF-κB	Nuclear Factor kappa-light-chain-enhancer of activated B cells
NO	Nitric oxide
OGTT	Oral glucose tolerance test
PAI-1	Plasminogen activator inhibitor-1
PBMCs	Peripheral blood mononuclear cells
PC-3	Prostate cancer cell line 3
PLA	Polylactic acid
PLE	Pressurized liquid extraction
PUFA	Polyunsaturated fatty acid
RBC	Red blood cells
ROS	Reactive oxygen species
SBP	Systolic blood pressure
SKOV-3	SK ovary 3
SFE	Supercritical fluid extraction
SOD	Superoxide dismutase
STZ	Streptozotocin
TC	Total cholesterol
TG	Triglyceride
TNF-α	Tumor necrosis factor alpha
TPC	Total phenolic content
UAE	Ultrasound-assisted extraction
UPLC-MS	Ultra-performance liquid chromatography-mass spectrometry
VLDL	Very low-density lipoprotein
WBC	White blood cells
